# Nanomotor-Assisted Intravesical Chemotherapy for Bladder Tumor Reduction and Suppression of Early Tumor Regrowth

**DOI:** 10.1021/acs.nanolett.5c05411

**Published:** 2026-04-23

**Authors:** Kristin Fichna, Maria Crespo-Cuadrado, Acsah Konuparamban, Valerio Di Carlo, David Esporrín-Ubieto, Ines Macías-Tarrío, Oriol Jutglar Soler, Shuqin Chen, María Gómez-Martínez, Anna C. Bakenecker, Antoni Vilaseca, Jordi Llop, Samuel Sánchez

**Affiliations:** † 284118 Institute for Bioengineering of Catalonia (IBEC), The Barcelona Institute for Science and Technology (BIST), Baldiri i Reixac 10-12, 08028 Barcelona, Spain; ‡ Doctorate in Biotechnology, Facultat de Farmàcia i Ciències de l’Alimentació, Universitat de Barcelona, Avda. Diagonal 643, 08028 Barcelona, Spain; § 90216 CIC biomaGUNE, Basque Research and Technology Alliance (BRTA), Paseo Miramón 182, 20014 Donostia/San Sebastián, Spain; ∥ Department of Urology, 16493 Hospital Clinic of Barcelona, 08036 Barcelona, Spain; ⊥ Catalan Institution for Research and Advanced Studies (ICREA), Passeig Lluís Companys 23, 08010 Barcelona, Spain; a Departament de Bioquímica i Fisiologia Facultat de Farmácia i CCAA, Universitat de Barcelona, Av. Joan XXIII, 27-31, 08028 Barcelona, Spain

**Keywords:** Nanoparticles, Nanomotors, Mitomycin C, Bladder Cancer, Drug Delivery, Intravesical Chemotherapy

## Abstract

Nanoparticles are widely used in nanomedicine for controlled drug delivery and improved bioavailability. However, their effectiveness is often limited by passive diffusion, especially in confined, fluid-filled environments like the bladder, where rapid drug clearance and uneven distribution reduce therapeutic impact. These challenges contribute to high recurrence in bladder cancer despite intravesical chemotherapy. To address this limitation, we present urease-powered nanomotors (NM) based on mesoporous silica nanoparticles loaded with Mitomycin C (MMC), the standard chemotherapeutic for nonmuscleinvasive bladder cancer. These NM useurea present in urine to induce motion and drug dispersion. *In vitro*, NM showed 2.3-fold higher uptake in mouse bladder cancer cells than passive nanoparticles and achieved the efficacy of free MMC (577.5 μg/mL) at a 20-fold lower dose (30 μg/mL). *In vivo*, a single intravesical dose reduced tumor volumes by 83% and prevented early tumor regrowth, demonstrating the potential of NM-based delivery for bladder cancer therapy.

## Graphical abstract

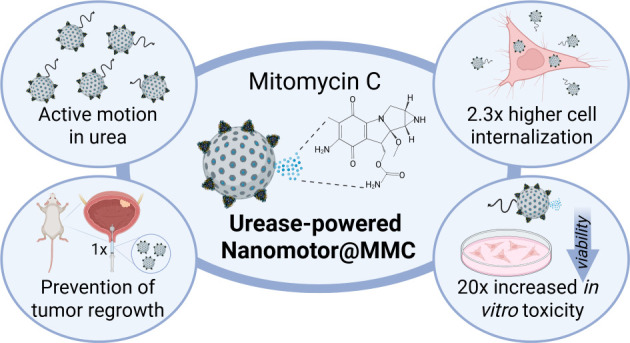

Nanotechnology has transformed the landscape of cancer treatment by enabling targeted drug delivery, enhanced bioavailability, and reduced systemic toxicity. A wide variety of nanocarrier systems have been developed to improve the efficacy of therapeutic agents through controlled release and site-specific accumulation. These nanoscale platforms aim to overcome the limitations of conventional treatments, by improving drug stability and tissue penetration.^1–3^

Despite these advancements, most nanoparticle-based systems still rely on passive diffusion for drug transport. This mechanism is inherently limited by biological barriers, uneven tissue perfusion, and physiological clearance mechanisms, ultimately leading to suboptimal drug accumulation at the target site.^4–7^ An emerging solution to these challenges lies in the development of self-propelled micro/nanoparticles (nanomotors, NM),^8–10^ which can actively navigate biological environments and overcome diffusion-limited drug delivery.^11–15^ NM can be powered by a variety of stimuli including magnetic fields,^16^ temperature gradients,^17^ ultrasound,^18^ or chemical reactions.^19–22^ Among them, enzyme-powered NM, particularly those powered by biocatalysts like urease, have gained increasing attention for their biocompatibility and autonomous motion without external control.^23–27^ These urease-powered NM convert urea, naturally occurring in urine,^28^ into ammonia and carbon dioxide, generating ionic gradients and convective flows that propel the NM.^29–33^

Bladder cancer represents a compelling target for this technology, being one of the most common cancers worldwide, with approximately 75% of cases diagnosed as nonmuscleinvasive bladder cancer (NMIBC).^34^ Standard treatment involves transurethral resection of the bladder tumor followed by intravesical immunotherapy and/or chemotherapy.^35^ However, NMIBC shows high recurrence rates (50–80% within five years),^36^ posing a persistent clinical and economic burden.^37^ Despite intravesical administration of Mitomycin C (MMC), the standard chemotherapeutic for intermediate-risk NMIBC,^38–41^ outcomes remain limited due to continuous urine production, which rapidly dilutes the drug and reduces its retention.^42–44^ To improve MMC delivery, various nanoparticle systems such as chitosan-poly-ε-caprolactone composites,^45^ RGD-decorated micelles,^46^ quantum dot-chitosan conjugates,^47^ and hyaluronic acid/mannitol particles^48^ have been explored, yet their efficacy remains constrained by passive diffusion, limiting penetration into residual or deeply embedded tumor cells. Using urease-powered NM therefore offers a unique opportunity to address these limitations. Previously, they have been shown to enable *in vitro* targeting of bladder cancer spheroids,^24^ intracellular gene delivery,^27^ and active motion *in vivo*.^13^ Additional studies have explored their use in localized radio-,^26^ photothermal-,^49^ and immunotherapy^50^ for bladder cancer. However, their application in delivering approved chemotherapeutics such as MMC remains largely unexplored, and their potential to prevent early tumor regrowth in bladder cancer has not yet been evaluated.

In this paper, we present urease-powered NM loaded with MMC, based on mesoporous silica nanoparticles (MSNP), known for their high drug-loading capacity, biocompatibility, and tunable surface properties.^51–53^ We assessed the effect of drug loading on NM propulsion and examined *in vitro* uptake and therapeutic efficacy in mouse bladder carcinoma cells (MB49) and in an orthotopic murine bladder cancer model. This work demonstrates the potential of NM-assisted chemotherapy not only to reduce tumor volumes but also their potential to suppress early tumor regrowth.

We synthesized MSNP following a modified sol–gel Stöber method.^54^ The resulting MSNP had a mean diameter of 549.8 ± 69.2 nm, as determined by scanning electron microscopy (SEM) (Figure 1b, c). Figure 1d shows the morphology of the silica-based nanoparticles, as observed by transmission electron microscopy (TEM). While TEM confirms the porous morphology of the nanoparticles, the mesoporous structure is further evidenced by the type IV nitrogen adsorption–desorption isotherm (Figure S1), which is characteristic of mesoporous materials,^55^ thereby confirming the successful preparation of MSNP. Brunauer–Emmett–Teller (BET) analysis revealed a surface area of 828.18 m^2^/g, with an average pore diameter of 2 nm (Figure 1e) calculated via the Barrett–Joyner–Halenda (BJH) method.^56,57^ The observed mesoporous structure and the reported pore diameter are crucial parameters for successful drug loading in the following steps.

**Figure 1:**
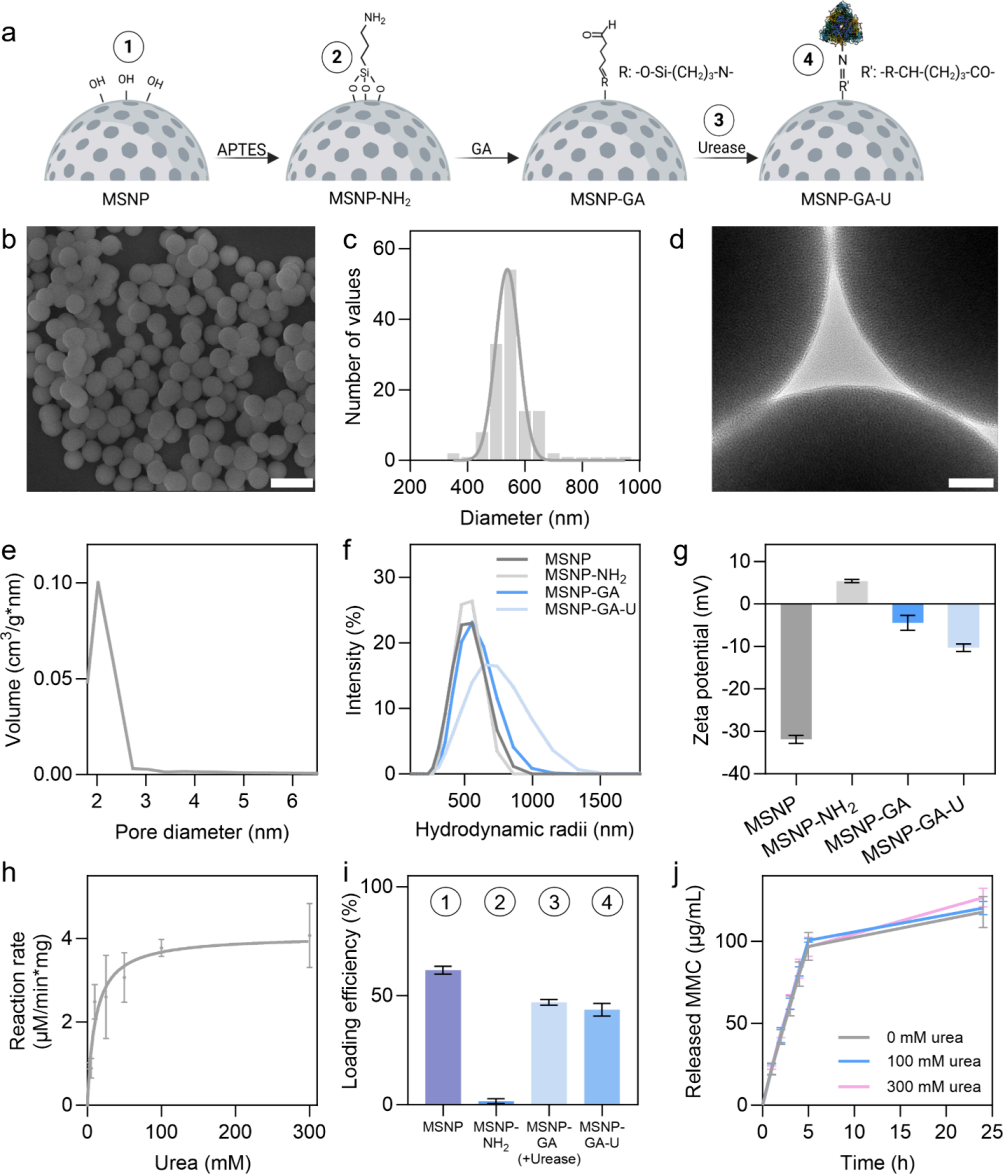
Synthesis and drug-loading of urease-powered NM. (a) Schematic illustration of surface modification of MSNP to obtain urease-powered NM. Created in BioRender. Sánchez, S. (2026) https://BioRender.com/crutn5v. (b) SEM microscopy image of MSNP. The scale bar corresponds to 1 μm. (c) Size distribution determined by SEM (*n* = 130). Particle size measurements were carried out using Fiji (ImageJ 1.54p, NIH) by measuring the diameter of individual particles. (d) TEM microscopy image of MSNP. The scale bar corresponds to 50 nm. (e) Pore size distribution of MSNP determined by BJH analysis applied to N_2_ isotherms data. (f) Hydrodynamic radii (nm) of MSNP at each functionalization step. (g) Surface charge evolution during surface modification of MSNP to obtain NM. (h) Michaelis–Menten fit for NM in the presence of different concentrations of urea. (i) Drug-loading efficiency of MMC at different steps of the functionalization of MSNP as indicated in the schematics. (j) Time-dependent cumulative release (μg/mL) of MMC from NM in the presence of different concentrations of urea (*n* = 3).

For the synthesis of urease-powered NM (Figure 1a), we functionalized MSNP with (3-aminopropyl)­triethoxysilane (APTES) to introduce surface amino groups through a reaction with surface hydroxyl groups. Subsequently, glutaraldehyde (GA) served as a bifunctional cross-linker to covalently bind urease to the particle surface, enabling asymmetric enzyme distribution required for self-propulsion.^29,58^ Dynamic light scattering (DLS) revealed increasing particle size, and changing surface zeta potential confirmed successful surface functionalization at each step (Figure 1f, g). MSNP exhibited a zeta potential of −31.8 ± 0.9 mV. After APTES modification, it shifted to 5.4 ± 0.36 mV, reflecting the successful introduction of positively charged amino groups. GA binding reduced the zeta potential to −4.44 ± 1.76 mV, and following urease conjugation, the zeta potential further decreased to −10.24 ± 0.9 mV. The enzyme’s isoelectric point is 5.0–5.2, making it negatively charged at neutral pH.^59^ The resulting negative zeta potential confirms the successful binding of urease to the nanoparticle surface. This was further supported by a bicinchoninic acid assay, quantifying an average of 81.70 ± 7.11 μg/mL of urease bond the NM which aligns with previous reports.^12,23,26^ The Michaelis–Menten fit revealed altered kinetic parameters for NM compared to those of the free enzyme (Figure 1h, Figure S2, Table S1) with a 2.3-fold decrease in *V*_max_ and a 3.1-fold increase in K_m_. These changes suggest a reduced catalytic rate and substrate affinity, most likely due to decreased accessibility of the enzyme’s active site caused by covalent binding to the nanoparticle.

Since the functionalization process to obtain NM involves multiple steps, we investigated at which stage MMC could be loaded (Figure 1a). As shown in Figure 1i, loading into MSNP (1) yielded the highest efficiency with 61.75 ± 1.80%, likely due to drug adsorption into the pores. Successful MMC loading was further confirmed visually by the light-blue color of the nanoparticles, reflecting the intrinsic color of the drug (Figure S5). In contrast, nonporous particles could not be loaded with MMC (Figure S4), highlighting the importance of a porous structure for drug loading. Loading into MSNP-NH_2_ (2) decreased the loading efficiency to 1.61 ± 1.06%, possibly due to electrostatic repulsion between amino groups of MMC and MSNP-NH_2_. Loading MMC into MSNP-GA (3) led to a loading efficiency of 46.96 ± 1.30%. For NM (4), it slightly decreased to 43.55 ± 2.88%, corresponding to 4.88 ± 0.6 mg MMC per mg MSNP. This decrease of 18% compared to step (1) may result from reduced pore accessibility due to the surface modifications. Since obtained loading efficiencies are in a similar range and to reduce potential interferences of the drug with the functionalization process, subsequent experiments were conducted using step (4) (Figure S6).

Our system offers a higher drug payload than previously reported methods, with estimated loading of 1.21 mg MMC/mg MSNP for similar techniques,^60^ 0.0625 mg MMC/mg nanoparticle using wet impregnation,^61^ and 0.127 mg MMC/mg nanoparticle for loading a lipidic prodrug into MSNP at 70 °C.^62^ To our knowledge, the example of MMC-loaded NM (NM@MMC) has not been reported before.

Drug release from NM@MMC was assessed in 0, 100, and 300 mM urea. Release profiles were similar across the urea concentrations (Figure 1j). Release of 100 μg/mL MMC occurred within the first 6 h, slowing between 6 and 24 h. After 24 h, 120 μg/mL were released per mg NM@MMC, which corresponds to 2% of the total drug loaded. Such a release profile from MSNP has been reported previously.^23^ Comparatively, we estimated that^60^ wet-impregnated MSNP released approximately 61.25 μg/mg within 16 min.^61^ Thus, our system achieves a slightly higher release of the loaded drug over time.

Urease catalyzes the decomposition of urea into ammonia and carbon dioxide, and when immobilized on the nanoparticle surface, this reaction generates ionic concentration gradients that enhance nanoparticle diffusion through self-diffusiophoresis.^12,23,31,63^

Using optical tracking (Movie S1), we evaluated the effect of drug loading on the single particle motion of NM in the presence of 0, 100, and 300 mM urea. Representative trajectories, mean square displacements (MSD), and diffusion coefficients of NM (Figure 2a) and NM@MMC (Figure 2b) were determined using a custom-made Python code. As shown in Figure 2a-ii and b-ii, MSD increased linearly over time, indicating diffusive motion with increased slopes at higher urea concentrations.^64,65^ Both NM and NM@MMC showed increased diffusion coefficients with rising urea concentration from 0.75 ± 0.04 to 0.95 ± 0.05 μm^2^/s for NM and from 0.71 ± 0.04 to 0.92 ± 0.06 μm^2^/s for NM@MMC (Figure 2a-iii and b-iii). No significant differences were observed for the diffusion coefficients of NM and NM@MMC (Figure S7), suggesting that MMC loading does not significantly impair the motion behavior at the single particle level. The obtained values align with previous reports on urease-powered NM, with diffusion coefficients ranging from 0.6 μm^2^/s (0 mM urea) up to 1.1 μm^2^/s in the presence of 300 mM urea.^23,24,66^

**Figure 2:**
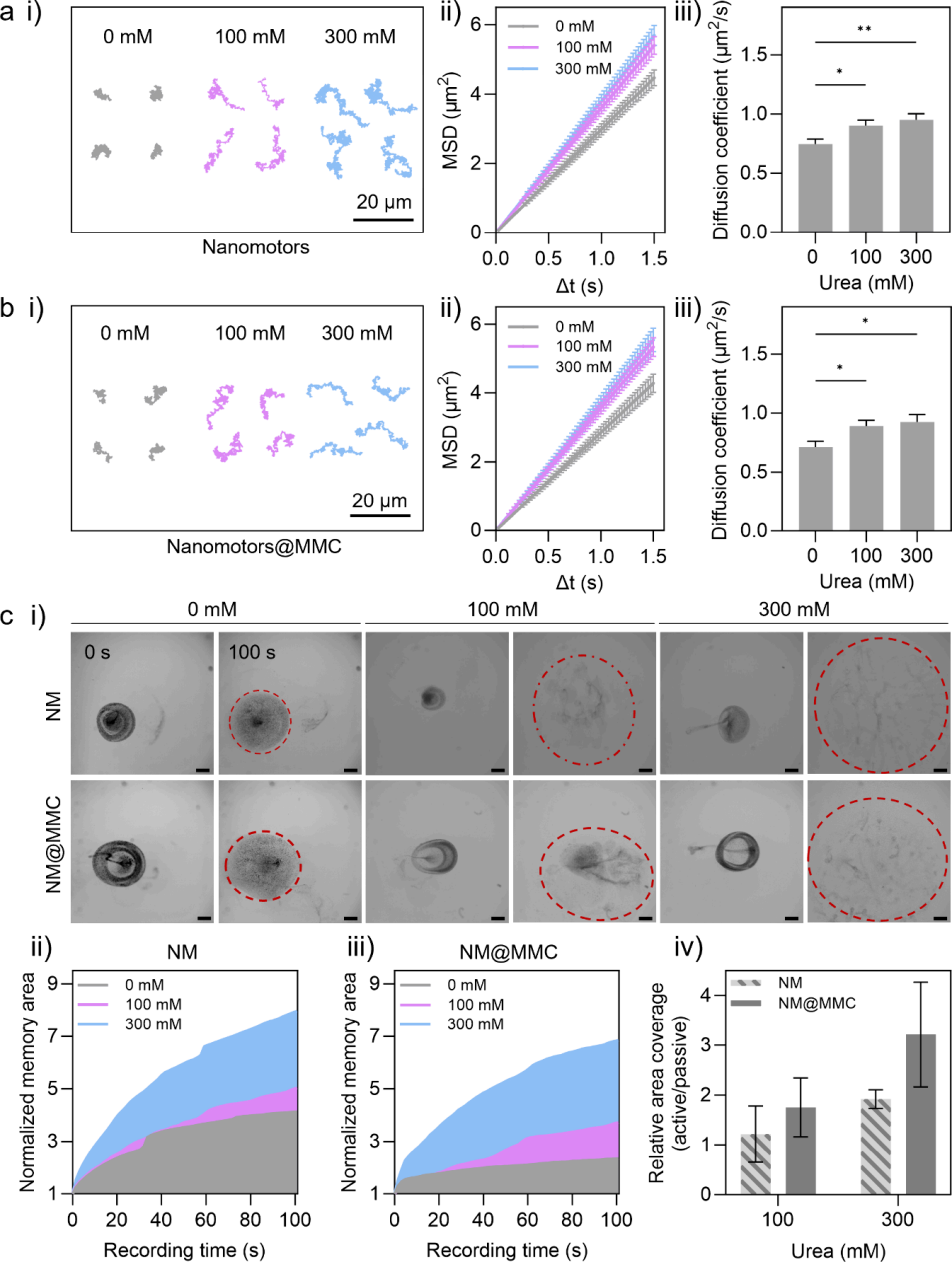
Motion analysis of (a) NM and (b) NM@MMC at single particle level. (i) Representative tracking trajectories during 30 s at relevant concentrations of urea, (ii) MSD, and (iii) effective diffusion coefficients. Statistical significance (one-way ANOVA) is indicated when appropriate (*p < 0.05, **p < 0.01, ***p < 0.001, ****p < 0.0001, *n* > 100 ± SE). (c) Collective motion analysis of NM and NM@MMC with (i) video snapshots in the presence of 0, 100, and 300 mM urea in PBS (scale bar corresponds to 1 mm), Normalized area (normalized to the initial area at *t* = 0 s) covered by (ii) NM and (iii) NM@MMC as a function of time and (iv) the relative covered area calculated by normalizing the area covered by NM and NM@MMC in 100 and 300 mM urea (*t* = 100 s) with the area covered in 0 mM urea (*t* = 100s).

The active motion of NM is accompanied by surrounding chemical changes, including the generation of ionic products and pH variations, which lead to buoyant effects and fingering instability, thereby exhibiting intriguing collective behavior.^30,67^ We further investigated the collective motion by placing a drop of NM or NM@MMC in the center of a PBS-filled Petri dish containing varying concentrations of urea and recording their motion over time (Movie S2 and Movie S3). In the absence of urea, the particles sedimented, whereas in its presence they spread across the dish (Figure 2c-i, highlighted with red circles). This fuel concentration-related collective behavior is consistent with our previously reported buoyancy-driven convection and diffusion, resulting from a density difference between the NM droplet, the urea-containing medium, and the reaction products.^30^ Quantitative analysis using a custom-made Python code (Figure S8) showed that the area covered by NM and NM@MMC (Figure 2c-ii, and c-iii) increased by 1.2- and 1.75-fold in 100 mM urea and by 1.9- and 3.2-fold in 300 mM urea, respectively, compared to 0 mM urea (Figure 2c-iv). Intensity profiles along the x- and y-axes (Figure S9) further confirmed this spreading behavior, in line with previous studies.^11–13^ Thus, NM@MMC demonstrated area coverage comparable to that of unloaded NM in the presence of urea.

To establish nontoxic NM and urea concentrations, we conducted *in vitro* tests on 2D mouse bladder cancer cell cultures (MB49), relevant to the orthotopic model.

First, we tested the toxicity of urea and NM separately. After 24 h, the cell viability assay showed no significant differences in relative fluorescence units (% RFU) between nontreated (NT) cells and those treated with 0–300 mM urea, confirming urea alone is nontoxic (Figure S10a, Figure S11). Similarly, NM concentrations from 0 to 25 μg/mL were well tolerated (Figure S10b).

Next, we combined NM and urea at different concentrations and assessed the percentage of live cells after 1 and 4 h using a live/dead cell viability assay (Figure S12). We estimated the percentage of live cells by calculating the ratio between the area covered by live cells in each sample, represented in green, and the area covered by live cells in the NT sample (Figure 3a). After 1 h (Figure 3a-i, a-ii), cells treated with 5 or 10 μg/mL NM in combination with up to 100 mM urea showed a high percentage of live cells (86.4–93.8%). However, 25 μg/mL of NM with 100 mM urea drastically reduced the percentage of live cells to 20.9%. All NM concentrations in combination with 300 mM urea caused high toxicity (1.18–2.2% viability), likely due to ammonia generated by the enzymatic reaction, which is known to be toxic at high concentrations in small volumes like *in vitro* setups.^23^ By increasing the incubation time to 4 h (Figure 3a-iii, a-iv), the percentage of live cells was maintained only in those samples incubated with 5 μg/mL NM combined with up to 100 mM urea, which correlates with results from the metabolic activity test (Figure S10c). To exclude a toxic effect of the generated subproducts at this condition, we incubated 5 μg/mL NM with 100 mM urea in cell medium to obtain the reaction subproducts after removing the particles from the solution. The cell viability remained high, 88.6% (Figure S13). Therefore, 5 μg/mL NM with 100 mM urea were selected for further experiments, excluding toxicity to MB49 cells coming from NM in the presence of urea, while ensuring self-propulsion with 100 mM urea.

**Figure 3:**
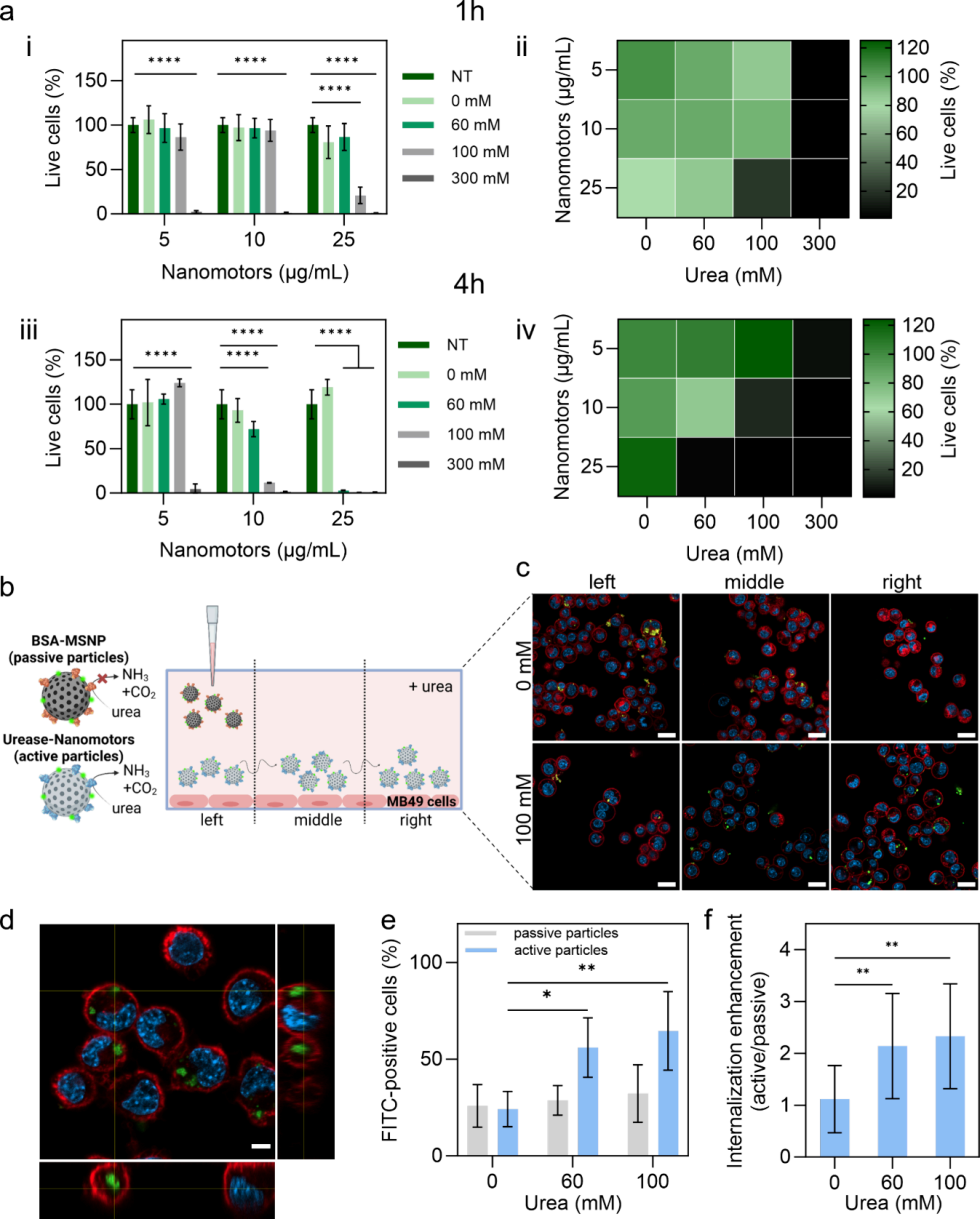
*In vitro* dosage optimization and cellular uptake of urease-powered NM. (a) MB49 cells were incubated with 5, 10, and 25 μg/mL of NM in combination with 0/60/100/300 mM of urea. Quantification plots of live cells (%) on fluorescence images (*n* = 3) and the corresponding heat-map of live cells (%) are shown for 1 h (i, ii) and 4 h (iii, (iv) of incubation. Statistical significance (two-way ANOVA) is indicated within groups when appropriate (*p < 0.05, **p < 0.01, ***p < 0.001). (b) Schematic representation of cell internalization study of FITC-labeled passive and active nanoparticles into MB49 cells. Created in BioRender. Sánchez, S. (2026) https://BioRender.com/t2b9k1v. (c) Confocal images showing MB49 cells with internalized FITC-labeled NM (green). The cell membrane has been labeled with WGA (red) and the nucleus with Hoechst stain (blue). The scale bar corresponds to 20 μm. (d) Orthogonal view of internalized NM in MB49 cells. Th scale bar corresponds to 5 μm. (e) Internalization efficiency of FITC-labeled particles into MB49 cells after incubating the cells for 1 h with 5 μg/mL particles and accessing the internalization rate by spectral flow cytometry after another 24 h. (f) Fold-enhancement of internalization rate of active over passive particles. Results are represented as mean ± SD (*n* = 4 biological replicates). Statistical significance (one-way ANOVA) is indicated when appropriate (*p < 0.05, **p < 0.01).

Next, using spectral flow cytometry, we investigated whether the active motion of NM increases cell internalization compared to passive particles. MB49 cells were incubated with 0/60/100 mM urea, and 3 μL of fluorescein isothiocyanate (FITC)-labeled NM or passive FITC-labeled MSNP functionalized with bovine serum albumin (BSA, Figure S14) were added to one side of the dish (Figure 3b), reaching a final concentration of 5 μg/mL. After 1 h, the medium was refreshed, and cells were incubated for 24 h. FITC-positive cells (Figure 3e) were then quantified by flow cytometry, gating on live cells, as shown in Figure S16, which presents the gating strategy and histogram analysis for the respective conditions. Passive particles exhibited similar levels of cell internalization (25–32% FITC-positive cells) across all tested urea concentrations, likely due to particle sedimentation (Figure S15). In contrast, NM internalization increased with urea concentrations, from 55.9% at 60 mM to 64.6% at 100 mM. With 100 mM urea, NM internalization was 2.33-fold higher than that for passive particles (Figure 3f). Confocal microscopy confirmed NM internalization into cells and distribution throughout the dish in the presence of urea (Figure 3c, d, Figure S17). Previously, a 1.2-fold increase of NM internalization in HeLa cells with 50 mM urea,^68^ a similar enhancement with PLGA-based NM in MB49 cells using 100 mM urea,^27^ and a 1.7-fold increase using enzyme-powered gold-NM in 4T1 cells after 2 h^69^ was reported. Therefore, our findings align with previous reports highlighting that active motion significantly enhances NM internalization.

To evaluate the therapeutic potential of our system, we conducted a live/dead cell viability assay on MB49 cells exposed to active NM@MMC under optimized conditions (5 μg/mL, 100 mM urea, 1 and 4 h treatment). Controls included NT cells, cells treated with free MMC (30 μg/mL corresponds to the amount of MMC loaded in 5 μg/mL NM), and NM@MMC without urea (passive particles). After 1 h, only active NM@MMC slightly reduced the percentage of live cells by 27.5%. After 4 h, this percentage dropped by 47.2%, while free MMC and passive particles showed minimal effects (Figure 4a, b). A metabolic activity assay confirmed these findings, with active NM@MMC reducing cell viability by 52.5% after 4 h (Figure 4c). To evaluate the therapeutic efficacy of our system, we determined the IC50 of free MMC in MB49 cells to be 577.5 μg/mL after 4 h of incubation (Figure S18). These results contrast with the 52.5% reduction in cell viability observed with active NM@MMC containing 30 μg/mL of MMC, leading to a 19.25-fold reduction in the effective dose compared to the free drug.

**Figure 4:**
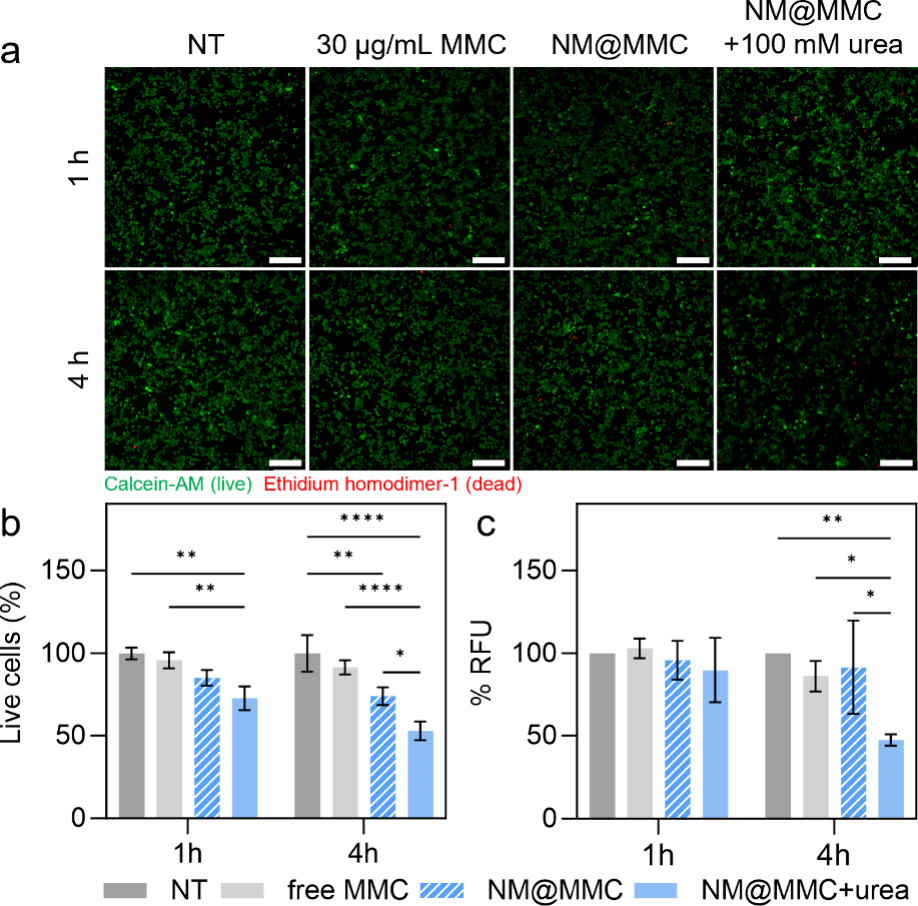
Therapeutic efficacy of NM@MMC in MB49 cells. (a) Live/dead images of NT cells and cells treated with free MMC (30 μg/mL, corresponds to amount of loaded MMC in 5 μg/mL of NM) and NM@MMC at 5 μg/mL in absence and presence of 100 mM urea. Images were taken after 1 and 4 h of incubation. The scale bar corresponds to 200 μm. Live cells are shown in green (Calcein-AM staining) and dead cells are shown in red (Ethidium homodimer-1 staining). (b) Quantification of live cells (%) based on live/dead images. (c) Metabolic activity (% RFU) of MB49 cells after 1 and 4 h of incubation with free MMC at 30 μg/mL, NM@MMC, and NM@MMC in the presence of 100 mM urea at 5 μg/mL. Metabolic activity has been determined using Presto Blue cell viability reagent. The fluorescence intensity has been normalized with the average fluorescence intensity of the NT cells to obtain %RFU. The results are represented as mean ± SD for (*n* = 3 biological replicates). Statistical significance (two-way ANOVA) within time points is indicated when appropriate (*p < 0.05, **p < 0.01, ***p < 0.001, ****p < 0.0001).

The need for reductive activation of MMC to cross-link DNA may explain the delayed toxicity despite fast NM internalization.^70,71^ Moreover, the lack of therapeutic effects of free MMC and passive NM@MMC supports that active motion enhances the drug delivery efficacy. Comparative studies, such as those by Pan et al., show a 2-fold increase in efficacy using active Janus-NM with Doxorubicin.^72^ Our group also reported a 10-fold improvement using urease-powered NM based on MSNP with doxorubicin in HeLa cells.^68^ Our findings demonstrate that active NM@MMC significantly improves drug delivery in MB49 cells, achieving up to a 2-fold improvement over previously reported systems, highlighting their potential for active drug delivery.

Next, we evaluated the therapeutic efficacy of NM@MMC *in vivo* using an orthotopic bladder cancer model in C57BL/6J female mice generated by intravesical instillation of MB49 cells. Tumor volumes were assessed via MRI 1 week (8–9 days) post-inoculation (Figure 5a). Mice with tumors >3 mm^3^ were randomly divided into four groups (n = 6), ensuring similar initial tumor volumes. MRI scans were performed at one week and two weeks post-treatment to monitor tumor progression (Figure 5b, Figure S21).

**Figure 5:**
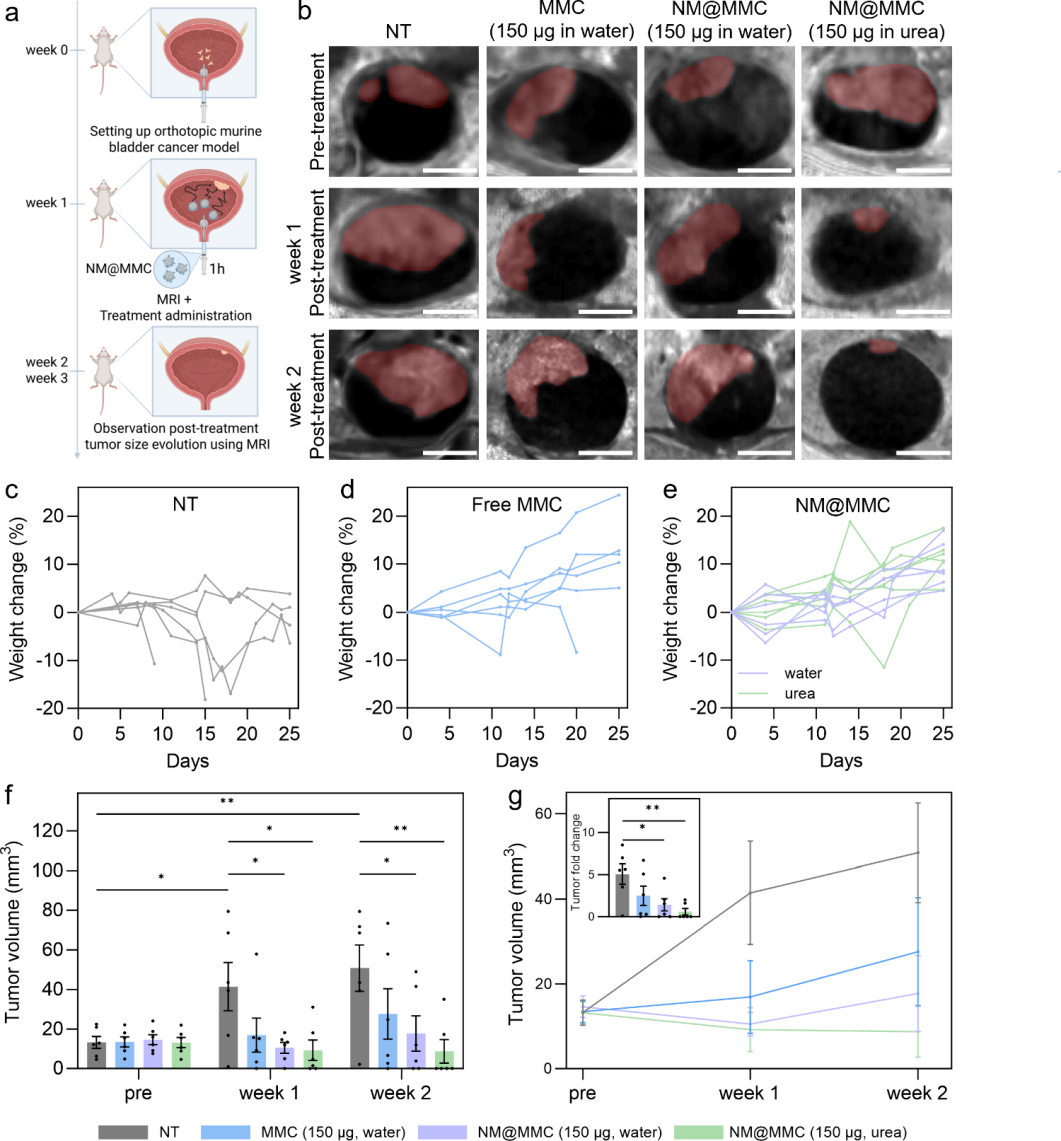
Therapeutic effect of NM@MMC in an orthotopic murine bladder cancer model. (a) Schematic illustration of timeline of intravesical chemotherapy using NM@MMC. Created in BioRender. Sánchez, S. (2026) https://BioRender.com/mxyb0os. (b) Representative 2D DW-MRI images of the bladder (hypointense circular region) of representative mice before and after treatment. Changes in body weight over time (*n* = 6 per group biological replicate, one line per animal) for (c) NT animals, (d) animals treated with free MMC, and (e) for animals treated with NM@MMC administered in water or urea. (f) Tumor volumes as determined using MRI before and after treatment. Results are expressed as bar diagrams (mean ± SE; one dot per animal). Statistical analysis was performed via two-way analysis of variance (ANOVA) followed by Tukey’s multiple comparison test. (g) Average tumor volume evolution over time (mean ± SE). Inlet: Tumor fold change after 2 weeks post-treatment. Results are expressed as bar diagrams (mean ± SE; one dot per animal). Statistical analysis for tumor fold change was performed via one-way analysis of variance (ANOVA) followed by Tukey’s multiple comparison test. Statistical significance is indicated when appropriate (*p < 0.05, **p < 0.01, ***p < 0.001, ****p < 0.0001).

In the NT group (Group 1), tumor volume increased notably from 13.2 ± 7.3 to 41.4 ± 29.8 mm^3^ at week one and 50.8 ± 28.7 mm^3^ at week two (tumor fold change 5.0 ± 2.9), with five out of six animals showing rapid tumor growth (Figure 5f, g). In Group 2 (free MMC, 150 μg), tumor progression was temporarily suppressed at week one (13.4 ± 6.3 to 16.9 ± 21.0 mm^3^), but regrowth occurred by week 2 (27.6 ± 31.14 mm^3^; fold change 2.5 ± 2.8).

Similarly, Group 3 (NM@MMC in water; 25 μg of NP, 150 μg of MMC) showed initial tumor reduction (14.6 ± 6.2 to 10.5 ± 6.7 mm^3^), but regrowth was observed by week two (17.7 ± 21.8 mm^3^; fold change 1.8 ± 1.8). In striking contrast, Group 4 (NM@MMC administered in 300 mM urea; 25 μg of NP, 150 μg of MMC) not only showed initial tumor volume reduction (13.2 ± 6.3 to 9.2 ± 12.7 mm^3^) but even continued regression after 2 weeks to 8.7 ± 14.6 mm^3^ (Figure 5g, fold change 0.6 ± 0.9). Furthermore, an 83% reduction in tumor volume compared to that in NT animals was achieved within this group (Figure S20), highlighting the critical role of nanoparticle motility for increasing retention times and thus improving therapeutic outcomes. Notably, compared to NT mice, intravesical treatment resulted in only minor weight fluctuations throughout the observation period (Figure 5c–e).

These findings demonstrate that active NM@MMC can go beyond short-term tumor suppression to prevent early tumor regrowth. While NM@MMC administered in water or free MMC showed only temporary effects, likely due to rapid drug clearance or poor tumor penetration, only NM@MMC administered in urea achieved durable tumor reduction. This sustained effect is likely due to enhanced tumor accumulation, improved drug delivery efficacy, and increased retention time.^13,26^ Overall, this study highlights the potential of self-propelled nanoparticles for bladder cancer therapy. NM@MMC, particularly when activated with urea, present a promising alternative to conventional intravesical MMC administration, offering enhanced drug efficacy, reduced early tumor regrowth, and improved long-term outcomes. Future studies exploring dosage optimization and extended monitoring will be key to advancing clinical translation.

In this work, we demonstrated that urease-powered NM based on MSNP significantly enhance the therapeutic efficacy of MMC for bladder cancer therapy. Initial *in vitro* studies optimized NM dosage, incubation time, and urea concentration, while ensuring mobility and effective cellular uptake, which was 2.3-fold higher than for passive particles. Compared to free MMC, NM@MMC achieved the same efficacy at a 19.25 times lower dose. *In vivo*, a single intravesical administration led to an 83% reduction in tumor volume and effectively prevented early tumor regrowth in an orthotopic murine bladder cancer model. Unlike free MMC, NM@MMC addressed major limitations in bladder cancer treatment, highlighting the potential of enzyme-powered NM for improving intravesical chemotherapy. Overall, these results open the way for using enzyme-powered NM to enhance the transport and therapeutic outcomes of approved drugs in oncology.

## Supplementary Material









## Notes

Characterization of NM@MMC and MSNP-BSA, calibration curve to determine MMC loading, loading efficiency of silica particles with MMC, enzymatic activity assays of urease, analysis of collective motion of NM and NM@MMC, dosage optimization of urease-NM in the presence of urea with MB49 cells based on live/dead cell viability assay and Presto Blue cell viability assay, toxicity of urea and subproducts of the reaction based on live/dead cell viability assay, flow cytometry gating strategies, confocal images showing internalized NM, and IC_50_ values of MMC in MB49 cells and live/dead images of MB49 cells treated with free MMC (DOCX)
Movie S1: Single particle tracking of NM and NM@MMC in the presence of 0/100/300 mM urea in PBS (MP4)
Movie S2: Collective motion of NM in the presence of 0/100/300 mM of urea in PBS (MP4)
Movie S3: Collective motion of NM@MMC in the presence of 0/100/300 mM of urea in PBS (MP4)

## Abbreviations

APTES: (3-Aminopropyl)­triethoxysilane
BET: Brunauer–Emmett–Teller
BJH: Barrett–Joyner–Halenda
BSA: Bovine serum albumin
CTAB: Cetyltrimethylammonium bromide
DLS: Dynamic light scattering
GA: Glutaraldehyde
FITC: Fluorescein Isothiocyanate
MMC: Mitomycin C
NMIBC: Nonmuscleinvasive bladder cancer
NM: Nanomotors
NM@MMC: Nanomotors loaded with Mitomycin C
MSD: Mean square displacement
MSNP: Mesoporous silica nanoparticles
NT: Nontreated
SEM: Scanning electron microscopy
TEA: Triethanolamine
TEM: Transmission electron microscopy
TEOS: Tetraethyl orthosilicate
RFU: Relative fluorescence units
