# Supplementary material for: Nanomotor-Assisted Intravesical Chemotherapy for Bladder Tumor Reduction and Suppression of Early Tumor Regrowth
Source: Nano Lett. 2026 Apr 23;26(17):5618–27. doi: 10.1021/acs.nanolett.5c05411 (PMC13154347; doi:10.1021/acs.nanolett.5c05411)
Supplement: Supplementary file 1 [file nl5c05411_si_001.docx]

**Supporting Information**

**Nanomotor-Assisted Intravesical Chemotherapy for Bladder Tumor Reduction and Suppression of Early Tumor Regrowth**

Kristin Fichna^1,2^, Maria Crespo-Cuadrado^1^, Acsah Konuparamban^3^, Valerio Di Carlo^1^, David Esporrín-Ubieto^1^, Ines Macías-Tarrío^1,2,4^, Oriol Jutglar Soler^1^, Shuqin Chen^1^, María Gómez-Martínez^3^, Anna C. Bakenecker^1^, Antoni Vilaseca^5^, Jordi Llop^3^, Samuel Sánchez^*,1,6^

^1^Institute for Bioengineering of Catalonia (IBEC), The Barcelona Institute for Science and Technology (BIST), Baldiri i Reixac 10-12, 08028 Barcelona, Spain

^2^Doctorate in Biotechnology, Facultat de Farmàcia i Ciències de l’Alimentació, Universitat de Barcelona, Avda. Diagonal 643, 08028 Barcelona, Universitat de Barcelona, Spain

^3^CIC biomaGUNE, Basque Research and Technology Alliance (BRTA), Paseo Miramón 182, 20014, Donostia/San Sebastián, Spain

^4^Departament de Bioquímica i Fisiologia, Facultat de Farmàcia i CCAA, Universitat de Barcelona, Av. Joan XXIII, 27-31, 08028-Barcelona

^5^Department of Urology, Hopital Clinic Barcelona, 08036 Barcelona, Spain

^6^Catalan Institution for Research and Advanced Studies (ICREA), Passeig Lluís Companys 23, 08010 Barcelona, Spain

**Methods and experimental details.**

**Materials.** Ethanol (EtOH, 99%), methanol (MeOH, 99%), hydrochloric acid (37% in water), ammonium hydroxide (25% in water), tetraethyl orthosilicate (TEOS, 99%), triethanolamine (TEOA, 99%), cetyltrimethylammonium bromide (CTAB, 99%), 3-amino-propyltriethoxysilane (APTES, 99%), glutaraldehyde (GA, 25% in water), urease (from Canavalia ensiformis, type IX, powder, 50 000−100 000 units per gram of solid), urea (99.9%), formaldehyde solution (37% in water), bovine serum albumin (lyophilized powder), dimethyl sulfoxide (DMSO, 99.9%), Phenol red, Fluorescein isothiocyanate isomer I and Silica nanoparticles (non-porous, 500 nm particle size, 5 % (w/w) in water) were purchased from Sigma-Aldrich. Pierce BCA Protein Assay Kit, Wheat Germ Agglutinin (WGA AlexaFluor 647 conjugate), phosphate-buﬀered saline (PBS), Live/dead viability/cytotoxicity Kit for mammalian cells and PrestoBlue for cell viability were purchased from Thermo Fisher Scientific. Hoechst 33342 was purchased from Life Sciences. DMEM low glucose medium, penicillin−streptomycin solution, fetal bovine serum (FBS) and trypsin 0.5% EDTA were purchased from Gibco. Mitomycin C (national code: 714183, Medac) was obtained from Hospital Clinic, Barcelona.

**Synthesis of mesoporous silica nanoparticles.** The MSNP (type MCM-41) were prepared using a modified Stoeber method. A solution containing CTAB (570 mg), TEOA (35 g), and Milli-Q H_2_O (20 mL) was heated and mixed at 95°C in a silicon oil bath and stirred for 30 min. Then, TEOS (1.5 mL) was added, and the mixture was further stirred for 2 h at 95ºC. The resulting particles were collected by centrifugation (1350 g, 15 min, 4ºC) and washed 3 times with EtOH abs. Then, the particles were resuspended in 30 mL of a MeOH:HCl mixture (10:0.6) and refluxed at 80 °C for 24 h, to remove the CTAB and open the MSNP pores. The particles were collected by centrifugation (1350 g, 15 min, 4ºC) and washed 3 times with EtOH abs, with 10 min sonication between washes. Then the CTAB removal was repeated one more time to properly open the pores of MSNP. Aliquots (0.5 mL) of MSNP were centrifuged and air-dried to determine their concentration by dry weighting. A drop of MSNP in EtOH abs was placed on top of a silicon wafer and scanning electron microscopy (SEM) images were taken using a FEI NOVA Nano SEM 230 at 10 kV to determine the particle size.

**Nitrogen adsorption-desorption.** The pore size distribution of MSNP was determined using nitrogen adsorption-desorption isotherms with a Micromeritics Tristar 3000 surface area and porosity analyzer. Nitrogen (N₂) was used as the adsorption gas. To ensure the accuracy of the analyzer, carbon black (Lot: 180049/180052) with a surface area of 21.3 ±0.75 m²/g was used as a certified reference material. Before the analysis, the samples were degassed at 100 °C for 18-20 h to remove any pre-adsorbed contaminants. The nitrogen adsorption measurements were carried out at relative pressures (P/P₀) of 0.10, 0.15, 0.20, 0.25, and 0.30 to ensure an accurate determination of the adsorption isotherm. From this, the surface area and pore size distribution have been calculated.

**Synthesis of FITC-labeled MSNP.** To obtain FITC-MSNP, a mixture of FITC (2 mg), EtOH (5 mL), and APTES (400 µL) was prepared and stirred for 30 min at room temperature. The MSNP synthesis protocol was followed as previously described except for the TEOS addition step, when TEOS (1.25 mL) was added in combination with the FITC-APTES mixture (250 µL). The reaction mixture was protected from light.

**Amine Functionalization of MSNP**. The synthesized MSNP (with and without FITC) were suspended at a concentration of 1 mg/mL in EtOH (70% in Milli-Q H_2_O) and mixed with APTES (6 µL/mg of MSNP). The solution was stirred at 70°C for 1 h. The particles were collected by centrifugation (1150 g, 5 min), and washed three times with EtOH and three times with Milli-Q H_2_O, with 5 min of ultrasonication before the next washing step.

**Functionalization of MSNP-NH_2_ with Urease.** MSNP-NH_2_ were centrifuged, resuspended in 900 µL of PBS 1× (1 mg/mL), and then sonicated for 5 min. Then, 100 µL of glutaraldehyde was added, and the mixture was placed on an end-to-end rotary shaker for 2 h at room temperature. Afterward, the nanoparticles were collected by centrifugation (1150 g, 5 min), and washed three times with PBS 1×, with 5 min of sonication between washes. After that, the resulting pellet was suspended in a 3 mg/mL solution of urease in PBS and placed on an end-to-end rotary shaker overnight at room temperature. The functionalized nanomotors were washed three times with PBS 1× by centrifugation (1150 g, 5 min) to remove excessive urease.

**Hydrodynamic Radii and Surface Charge Analysis.** Dynamic Light Scattering (DLS) was used to determine the hydrodynamic radii, size distribution, and surface charge of the Nanoparticles a Zetasizer Nano ZS (Malvern Instrument). Nanoparticles were analyzed after each functionalization step: MSNP, MSNP-NH2, MSNP-GA and MSNP-GA-Urease (NM). For each condition, 3 replicates were acquired.

**Quantification of Urease and BSA amount bound on the MSNP.** The concentration of urease and BSA bound on the MSNP’ surface was measured using Pierce BCA Protein Assay Kit following the manufacturer’s instructions. 25 µL of the supernatants collected during the washing steps of the nanomotors were added to a 96-well plate, together with the stock solution of urease or BSA solution that has been previously used for the functionalization. Samples were mixed with 200 µL of a working solution of Reagent A and B (50:1) and incubated for 30 min at 37 °C. Absorbance was read at 562 nm using a SYNERGY HTX absorbance microplate reader. The concentration of urease or BSA on the particles was calculated by interpolating absorbance values to a BSA standard plot and using equation (1):

$$protein bound \left( \frac{\mu g}{mL} \right)$$

$$=stock concentration \left( \frac{\mu g}{mL} \right)-concentration in supernatant \left( \frac{\mu g}{mL} \right) (1)$$

**Mitomycin C-loading of nanomotors.** 1 mg/mL MSNP, MSNP-NH2, MSNP-GA+Urease or MSNP-GA-urease (NM) have been collected by centrifugation (1150 g, 5 min). After removing the supernatant, the nanoparticles were resuspended in 1 mL of 10 mg/mL MMC solution in PBS 1x and incubated overnight in an end-to-end rotary shaker protected from light. After the overnight incubation, nanoparticles have been washed 3x with PBS and the supernatants have been collected for determining the MMC concentration in the supernatants after each washing step. MMC concentrations have been determined by measuring the absorbance values of the supernatants at 360 nm using a SYNERGY HTX absorbance microplate reader. The MMC concentration has been determined using a calibration curve (Figure S 3) following the Lambert-Beer law. Then, the amount loaded has been calculated following equation (2):

$$MMC loaded \left( \frac{mg}{mL} \right)$$

$$=stock concentration \left( \frac{mg}{mL} \right)-concentration in supernatant \left( \frac{mg}{mL} \right) (2)$$

The loading efficiency has been calculated as follows:

$$Loading efficiency \left( \% \right)= \frac{{MMC}_{loaded}\left( \frac{mg}{mL} \right)}{stock concentration \left( \frac{mg}{mL} \right)}*100\% (3)$$

**Mitomycin C release from urease-nanomotors.** Urease-nanomotors have been loaded as described above. After successful loading, nanomotors (1 mg/mL) have been resuspended in 0/100/300 mM urea in PBS 1x and were incubated in a Thermomixer at 37ºC. After every hour, the nanoparticles have been centrifuged to collect the supernatants. Then, the particles have been resuspended in fresh urea solution and placed back in the thermomixer. Supernatants have been collected after 1/2/3/4/5/24 h of incubation and concentration of MMC in the supernatants has been determined as described above. The amount MMC released has been calculated as follows:

$${MMC}_{released}\left( \frac{\mu g}{mL} \right)=\frac{Abs Supernatant \left( 360 nm \right)-0.0426}{1.9826}*Dilution factor*1000 (4)$$

The cumulative release has been calculated as follows:

$$MMC released \left( \% \right)=100-\left( \frac{{MMC}_{loaded}-{MMC}_{released}}{{MMC}_{loaded}}*100\% \right) (5)$$

**Enzymatic activity assay.** The enzymatic activity of free urease and urease-nanomotors w/o drug has been measured by monitoring the pH change induced by urease during the conversion of different concentrations of urea over time using Phenol Red (0.025 mM, pH 6.5 in PBS) as pH indicator. For this, 2 µl of nanomotors and 200 µL of phenol red containing urea have been mixed and the pH change has been monitored over time (2 h, 37ºC) measuring absorbance at 560 nm. The slopes of the curves (Abs/s) have been determined and converted to the reaction rate (µmol/min) using the extinction coefficient of phenol red (31,620 M^-1^cm^-1^). The Michaelis-Menten curves have been obtained by testing different concentrations of urea (0-300 mM, Figure S 6).

**Single-Particle Tracking and Motion Analysis.** An inverted Leica DMi8 microscope coupled with a Hamamatsu high-speed camera and a 63× water-immersive objective was used to record the videos of the nanomotors at the single-particle level. A range of urea concentrations in PBS (0, 200, 600 mM) were placed on a glass slide (5 µL) and mixed 1:1 with the nanomotors (5 µL, 0.1 mg/mL). Samples were covered with a glass coverslip to avoid artifacts caused by drift, and videos were recorded for 60 s at a framerate of 40 FPS in bright field mode and a spatial resolution of 0.0636 μm/px. Data processing was performed in two main stages: background removal and single-particle tracking. To remove static background noise, each frame of the video was corrected by subtracting the average intensity image, computed from 100 randomly selected frames:

$$I_{corrected}\left( x,y,f \right)= I_{raw}\left( x,y,f \right)-\frac{1}{100}\sum_{k=1}^{100} I_{k}\left( x^{'}, y^{'} \right) (6)$$

where 𝐼_𝑟𝑎𝑤_(𝑥, 𝑦, 𝑓) is the intensity at pixel (𝑥, 𝑦) in frame 𝑓 of the video sequence, 𝐼_𝑘_(𝑥′, 𝑦′) is the intensity at pixel (𝑥 ′, 𝑦′) in the 𝑘 𝑡ℎ selected frame, and 𝐼_𝑐𝑜𝑟𝑟𝑒𝑐𝑡𝑒𝑑_(𝑥, 𝑦, 𝑓) is the resulting background-free frame. Following preprocessing, particle trajectories were extracted with a custom-made Python code, based on the open-source Trackpy library^[1]^. From these trajectories, the mean squared displacement (MSD) was calculated as:

$MSD \left( \Delta t \right)=\left\langle\sum_{i=1}^{n} (x_{i}\left( t+\Delta t \right)-x_{i}\left( t) \right)^{2} \right\rangle$ (7)

where 𝑡 is the time in seconds, and n=2 for 2D analysis. Finally, the diffusion coefficient (𝐷𝑐) [µ𝑚^2^ 𝑠 ^−1^] was then extracted from fitting the MSD curve to:

$$MSD \left( \Delta t \right)=4D_{c}\Delta t (8)$$

where 𝐷𝑐 is the diffusion coefficient. The error bars correspond to the 95% confidence interval (CI95%).

**Swarming behavior and collective motion analysis.** An inverted Leica DMi8 microscope coupled with a Hamamatsu high-speed camara and a 1.25× objective was used to record the videos for the *in vitro* collective motion behavior of nanomotors. A 35 mm Ibidi dish was filled with 3 mL of a range of urea concentrations in PBS (0, 100, 300 mM) and placed in the microscope. A 3 µL-drop of the nanomotors (10 mg/mL) was added to the middle of the dish and videos were recorded for 2 min at a frame rate of 25 FPS under bright field mode.

For calculating the cumulative area of the nanomotors swarms, first, the background was subtracted from each frame of the video. As a background image, a snapshot was taken before the nanomotors were added to the urea solution. Then, an intensity threshold was applied for all videos. Pixels of intensity values above this threshold are considered as the area occupied by the NM swarm. By using this threshold the video is converted into binary images. The known resolution of the microscope then allows us to convert the number of pixel into units of area.

The x and y projections were obtained by counting the number of pixels with an intensity value different than 0 for each x and y value.

**Cell Culture.** MB49 cells (murine urothelial carcinoma cell line) were cultured in Dulbecco’s modified Eagle’s medium (DMEM) containing D-glucose, L-glutamine, and sodium pyruvate. Full cell medium was prepared by adding 10% FBS and 1% penicillin-streptomycin as supplements. Cells were cultured at 37ºC and 5% CO_2_ atmosphere. Cells were split every second day, passing 2 million cells in a T75 flask to keep them in culture. Cells were used between passage 15-20 in each experiment.

**Fluorescence imaging MB49 cells after treatment with nanomotors.** MB49 cells were seeded at 50 000 cells/well in a 24-well plate 48 h prior to the experiments. To study biocompatibility of nanomotors, 1 mL of nanomotors at 5, 10 and 25 µg/mL have been added in a combination with different concentrations of urea (0-300 mM) in medium and incubated at 37ºC for 1 and 4 h. After that, cells have been washed with PBS and stained using LIVE/DEAD cell viability/cytotoxicity kit (invitrogen) according to the instructions of the manufacturer. Live and dead cells have been imaged using a THUNDER Leica microscope with a 10× objective equipped with the according filter cubes (Calcein AM: λ_ex_=494 nm, λ_em_=517 nm, Ethidium homodimer I: λ_ex_=528 nm, λ_em_= 617 nm). To study therapeutic efficiency of nanomotors containing drugs, 5 µg/mL of nanomotors w/o 100 mM urea have been tested accordingly. Furthermore, toxicity of free Mitomycin at 0-1500 µg/mL (1h, 4h, 24h of incubation); 100 mM urea (4h of incubation) and subproducts of the reaction (4h of incubation) have been studied accordingly.

**Metabolic activity of MB49 cells.** MB49 cells were seeded at 50 000 cells/well in a 24-well plate 48 h prior to the experiments. To assess the toxicity of urea (0-300 mM) and nanomotors (5-25 µg/mL) cells were incubated for 24 h with the according concentrations (final volume 1 mL). To access changes in the metabolic activity, PrestoBlue cell viability reagent was prepared as recommended by the manufacturer, at 1:10 ratio in PBS 1x, 1 mL of the reaction mixture was added to each sample and incubated for 10 min at 37ºC. After that, the fluorescence intensity has been measured (λ_ex_=560 nm, λ_em_=590 nm) in triplicates for each sample using a SYNERGY H1M fluorescence plate reader. The toxic effect of nanomotors (5, 10 µg/mL) in combination with increasing concentrations (0-100 mM) of urea has been evaluated accordingly, using 1h and 4h of incubation time. After evaluating biocompatible ranges of nanomotors in combination with urea, the therapeutic effect of drug-loaded nanomotors has been tested accordingly using 5 µg/mL nanomotors and 100 mM urea (1h, 4h of incubation). Furthermore, the toxicity of the subproducts of the reaction after 4 hours exposure has been tested accordingly.

**IC50 evaluation of free Mitomycin C.** MB49 cells were seeded at 50 000 cells/well in a 24-well plate 48 h prior to the experiments. To assess the concentration of free Mitomycin C required to inhibit MB49 viability, cells were incubated for 1, 4 and 24h with concentrations of 0 to 1500 µg/mL Mitomycin C. After the incubation (1h, 4h, 24h), metabolic activity was studied using PrestoBlue cell viability reagent as described previously. IC₅₀ values were calculated by fitting the data to a four-parameter sigmoidal (logistic) nonlinear regression model using GraphPad Prism.

**Flow cytometry.** MB49 cells were seeded at a density of 100k cells/well of a 6-well plate and incubated for 24 h at 37 °C, 5% CO_2_ prior to treatment. Before the experiments, cells were washed once with PBS 1x. After that, the dish was filled with 3 mL of cell medium w/o 60/100 mM urea. A 3 µL drop of FITC-labelled nanomotors or BSA-MSNP (10 mg/mL, final concentration in dish 5 µg/mL) has been injected into the left side of the dish. After incubation for 1h at 37ºC, the liquid has been removed, and a new medium has been added. After 24 h, cells have been washed two times and trypsinized (500 µL trypsin/well). To stop the trypsinization, 800 µL of full medium have been added. The single cell suspension has been transferred to a 1.5 mL tube and centrifuged 5 min at 300 g. The supernatant has been removed, and cells have been washed one more time with PBS (500 µL). Afterwards, cells have been resuspended in 200 µL PBS 1x containing DAPI (1:100 in PBS 1x). Control samples have been resuspended in PBS only. 100 µL of each single cell suspension has been transferred to a 96-well plate with round bottom for subsequent analysis by flow cytometry. Quantifications based on fluorescence were performed using a 4-laser spectral Aurora flow cytometer (Cytek). The resulting flow cytometry data was analyzed using FlowJo (Treestar,Inc., Ashland) software.

**Confocal imaging.** MB49 cells were seeded in a confluence of 50k cells/well in a 35 mm high µ-Dish (Ibidi) and incubated for 24h at 37ºC and 5% CO_2_. Subsequently, 3 mL of media w/o urea 100mM were added to the dish followed by a 3 µL drop of FITC-labeled NMs which were added on one side of the dish (left), resulting in a final concentration of 5 µg/mL of NMs in the total volume. After 1h treatment at 37º, the media was removed, and 3 mL of fresh media were added to the dish. After 24h, cells were washed with PBS (1 mL) and incubated with a staining solution containing Hoescht (1 µg/mL) and WGA (1 µg/mL) for 30 minutes. Cells were washed again with PBS (1 mL), and images were captured on different areas of the dish using a Zeiss LSM 800 confocal microscope with a 40x objective.

**Animal experiments: General considerations**

All animal procedures were conducted in accordance with the European Council Directive 2010/63/UE and internal guidelines. The study was approved by the CIC biomaGUNE ethics committee and local authorities (authorization code: PRO-AE-SS-184). The orthotopic murine model of bladder cancer was established via intravesical administration of MB49 cells to C57BL/6J female mice, following a previously described protocol.^[2]^

**Therapeutic Experiments**

Thirty-two female C57BL/6J mice (Charles River Laboratories, France; 6 weeks old) were inoculated with MB49 cells. Mice were anesthetized using 4% isoflurane in pure O₂ for induction and maintained with 1.0–2.0% isoflurane in 100% O₂. The bladder was emptied by abdominal massage, and chemical lesions were induced in the urothelium through the intravesical instillation of 50 µL poly-L-lysine (Sigma-Aldrich) using a 24-gauge catheter. The solution was retained for 15 minutes, after which the bladder was emptied again. Subsequently, 10⁶ MB49 cells/mL suspended in 100 µL of high-glucose DMEM were instilled into the bladder via the catheter and retained for 1 hour. The catheter was then removed, and the bladder was emptied through abdominal massage. Animals were returned to their cages and monitored during recovery from anesthesia.

Approximately one week (8-9 days) post-inoculation, tumor size was determined via magnetic resonance imaging (MRI) using a 7T Bruker BioSpec USR 70/30 scanner (Bruker BioSpin) equipped with a BGA-12S gradient insert of 440 mT m⁻¹. Mice were anesthetized with 4% isoflurane for induction and 1.5% isoflurane for maintenance in 100% O₂ and positioned on an MR-compatible cradle. Body temperature and respiration rate were continuously monitored using an MR-compatible monitoring device (Small Animal Instruments, model 1030 SA) interfaced with a small-rodent heating system. MRI images were acquired using a spin-echo-based diffusion-weighted imaging sequence with the following parameters: Echo time (TE): 22.3 ms; repetition time (TR): 2,000 ms; averages: 2; gradient duration (δ): 4.5 ms; gradient separation (Δ): 10.6 ms; field of view: 12 × 12 mm²; image matrix size: 160 × 160 points; slice thickness: 0.5 mm (20 consecutive slices, no gap, acquired in interleaved mode); bandwidth: 192.9 Hz per pixel

Tumors were visualized by post processing images in ImageJ software. Diffusion-weighted images (b = 650 s mm⁻²) were divided by basal images (b = 0 s mm⁻²), followed by the application of a 3D Gaussian filter (σx = σy = σz = 0.7). Tumors were manually delineated to determine their volume. Only mice with tumor volumes >3 mm³ were included in subsequent therapeutic studies, as smaller tumors at this stage often regress spontaneously over time (unpublished observations). The remaining 24 animals were evenly distributed into four groups (groups 1–4) to ensure homogeneity in average tumor volumes and standard deviations across groups.

Three to five days after the first MRI session, treatments were administered intravesically following the same protocol as described for cell inoculation. The treatment groups were as follows: Group 1 (Control): 100 µL PBS solution; group 2 (MMC): 100 µL PBS solution containing 150 µg of MMC; group 3 (NM@MMC, water): 100 µL of solution containing 25 µg of NM@MMC in water, corresponding to 150 µg total MMC. Group 4 (NM@MMC, urea): 100 µL of 300 mM urea solution containing 25 µg of NM@MMC, corresponding to 150 µg total MMC.

Following treatment, animals were monitored during recovery from anesthesia and returned to their cages. Body weight was recorded every second day. A human endpoint was applied if the animal lost more than 20% of its initial weight or based on clinical symptoms, as per the criteria of the veterinarian in charge. Seven to nine days after treatment, tumor sizes were reassessed using MRI, following the same imaging and postprocessing protocols described above. Values were expressed as total tumor volumes, or as tumor fold change, calculated as the ratio between tumor volumes after and before treatment.

**Statistical analysis.** All data are shown as the mean ± standard deviation, if not indicated otherwise. For *in vivo* studies, all data points are included in the bar diagrams. Statistical differences were analyzed using GraphPad Prism 8 software (La Jolla, CA). The according statistical test used in each experiment is stated in the figure caption below. Significance is considered with a p < 0.05(*), p < 0.01 (**), p < 0.001 (***), and p < 0.0001 (****).

**Supplementary Figures and Tables**


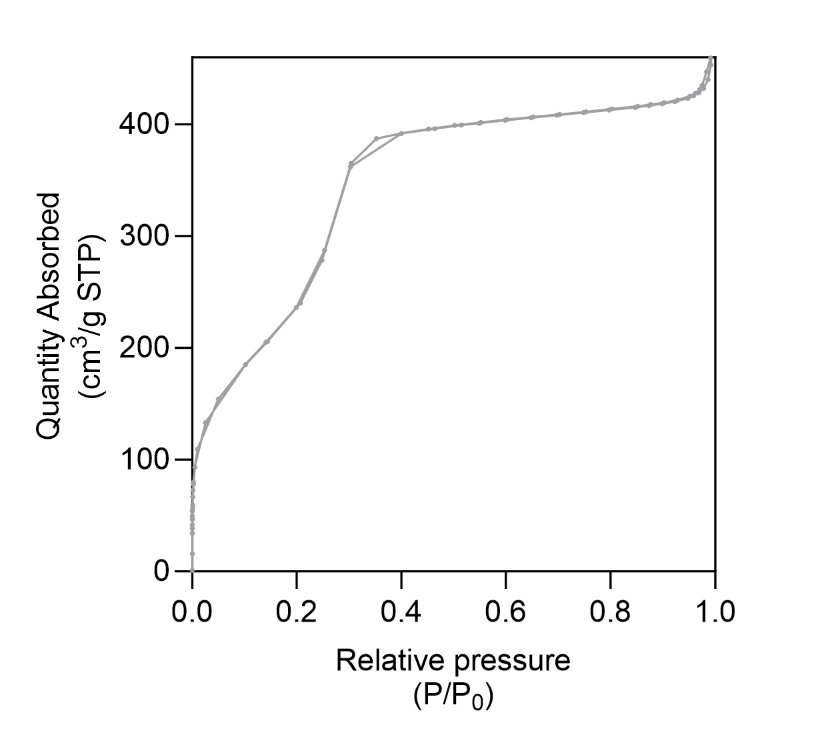


Figure S 1. N_2_ Adsorption/Desorption isotherm linear plot of MSNP nanoparticles.


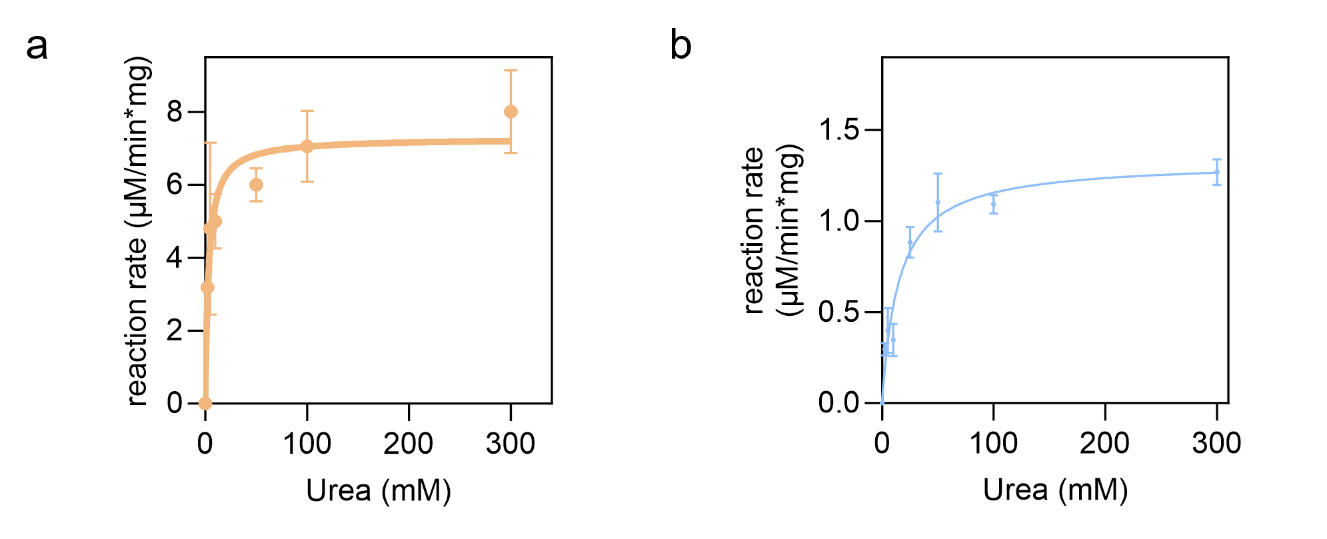


Figure S 2. Michaelis-Menten kinetics of a) free urease and b) NM@MMC in presence of urea.

Table S 1. Michaelis-Menten kinetics constants.

| sample | Vmax (µmol/min) | K_m_ (mM) | K_cat_ (s^-1^) |
| --- | --- | --- | --- |
| Free urease | 9.6 | 3.558 | 2.90E+05 |
| NM | 4.072 | 10.9 | 2.16E+05 |
| NM@MMC | 1.329 | 14.93 | 1.12E+05 |


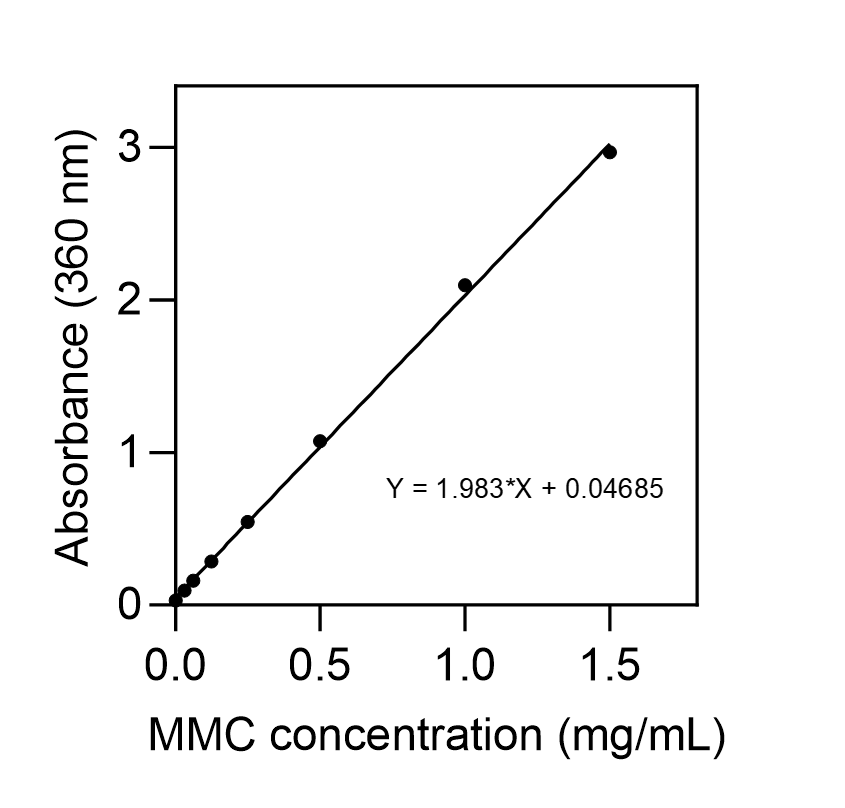


Figure S 3. Calibration curve of MMC in PBS. Absorbance has been determined at 360 nm.


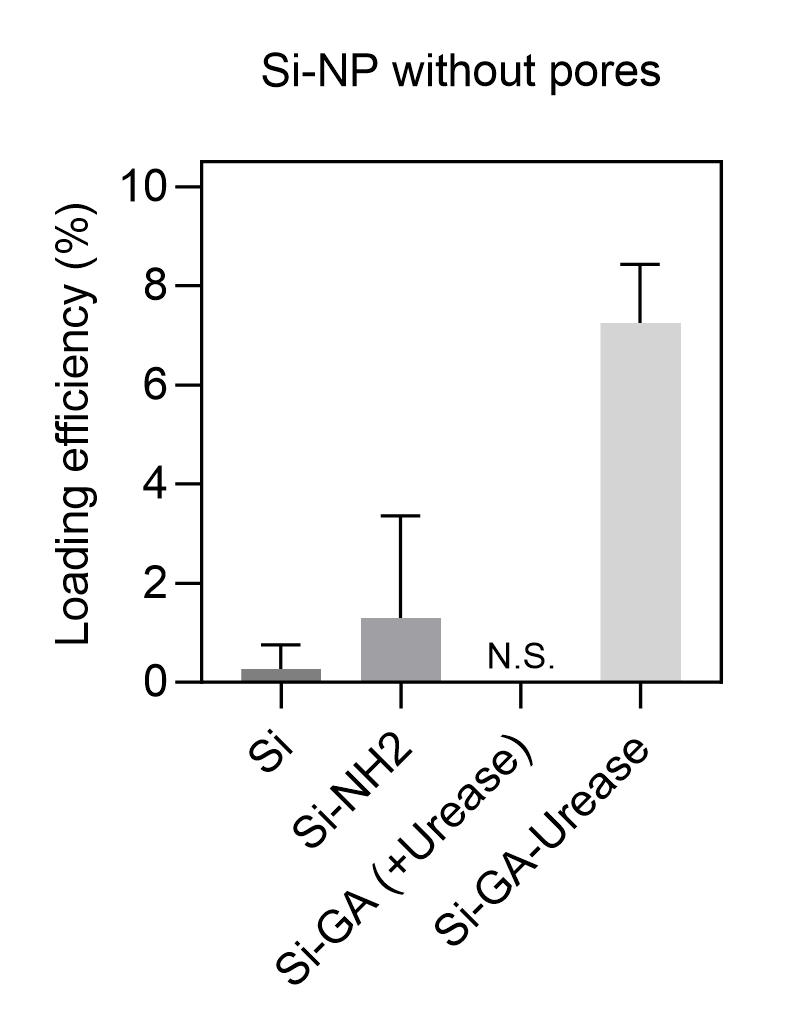


Figure S 4. The loading efficiency of MMC loaded into non-porous silica nanoparticles (500 nm). Silica nanoparticles have been functionalized similarly to MSNP and loaded with MMC at each functionalization step. (n=3)


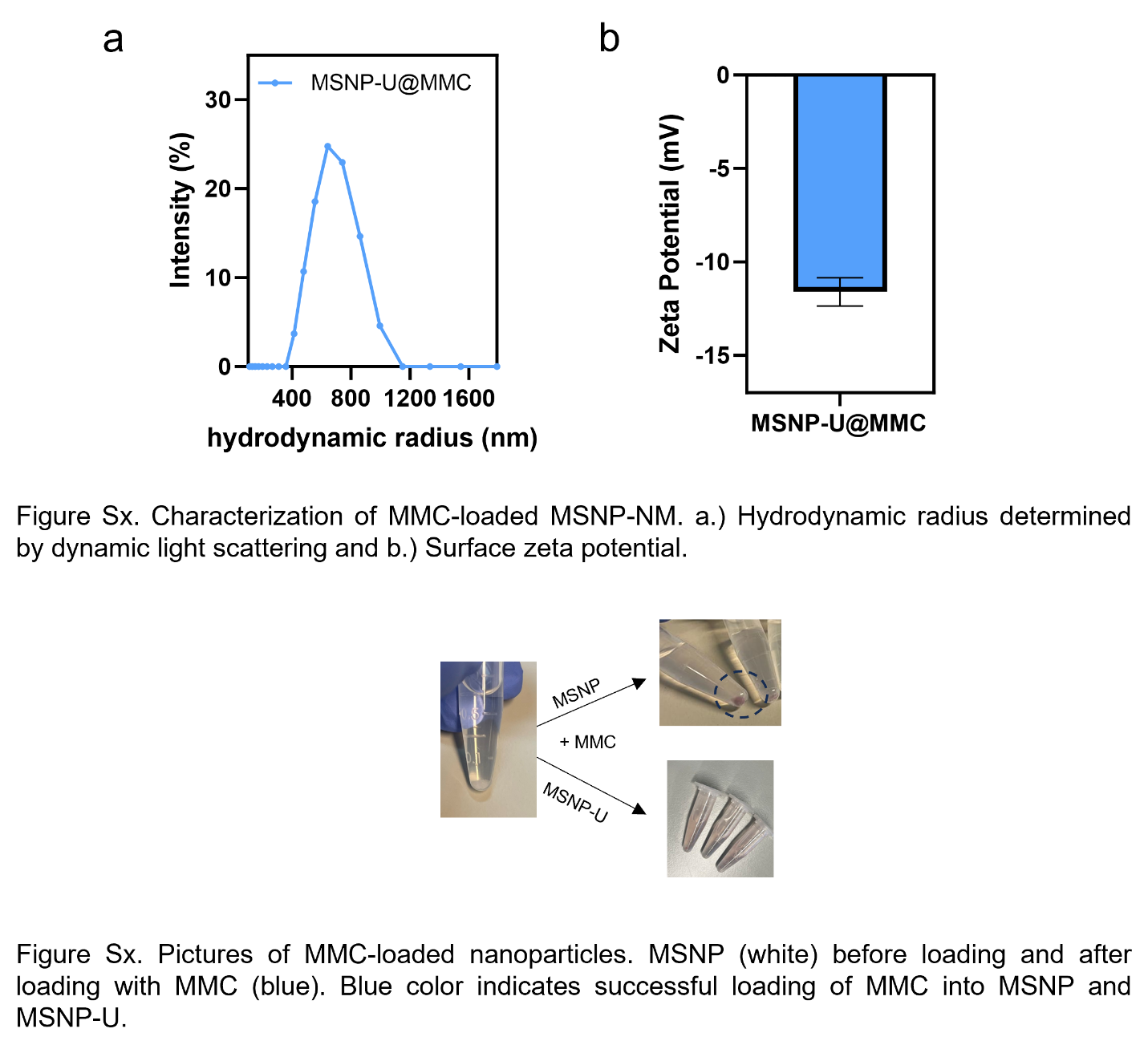


Figure S 5. Pictures of MMC-loaded nanoparticles. MSNP before loading and after loading with MMC (blue). Blue color indicates successful loading of MMC into MSNP or MSNP-U.


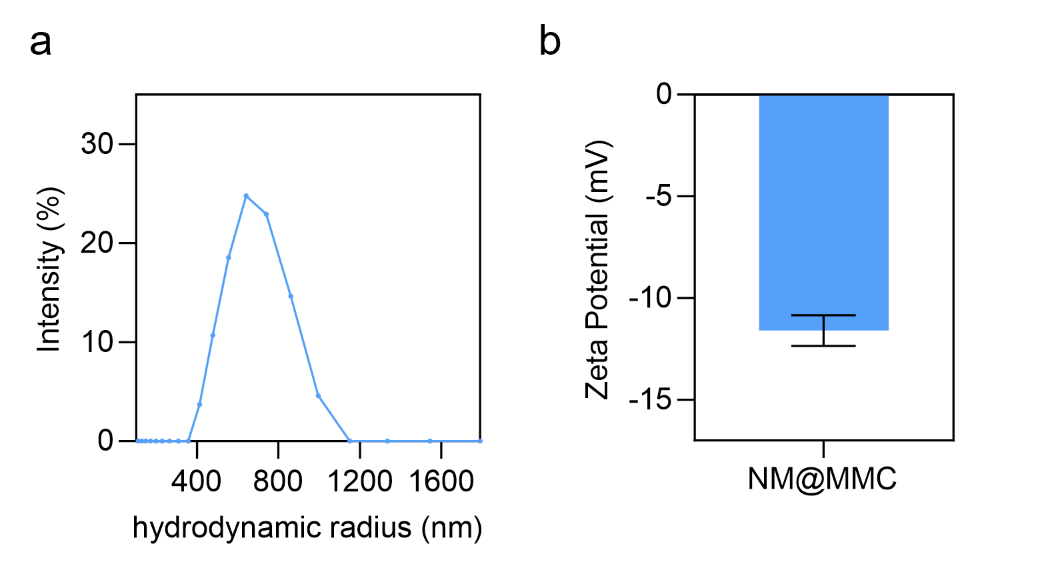


Figure S 6. Characterization of NM@MMC. a.) Hydrodynamic radius determined by DLS and b.) Surface zeta potential.


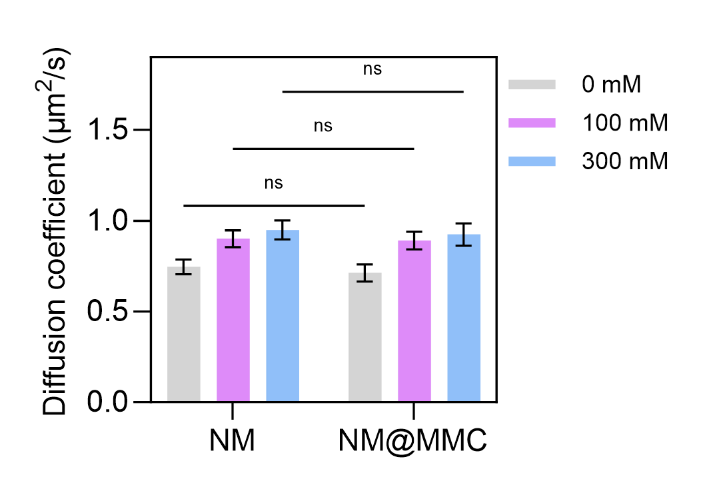


Figure S 7. Diffusion coefficient of NM and NM@MMC obtained by optical tracking at different urea concentrations.

**
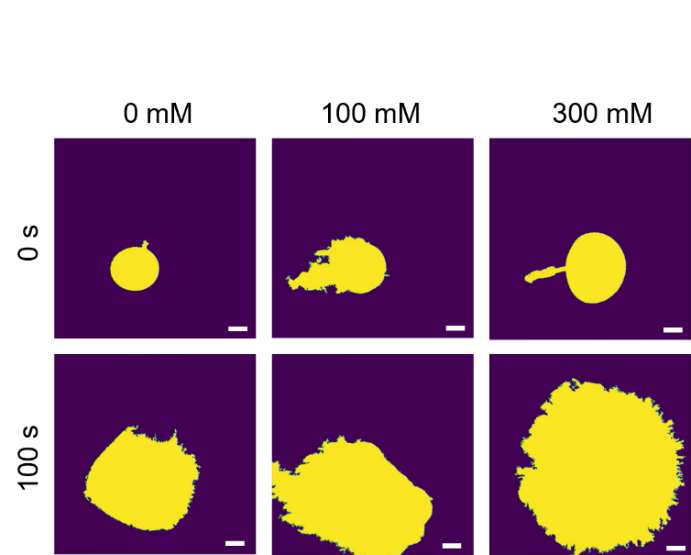
**

Figure S 8. Color coded videos for NM in 0/100/300 mM urea using a memory function.


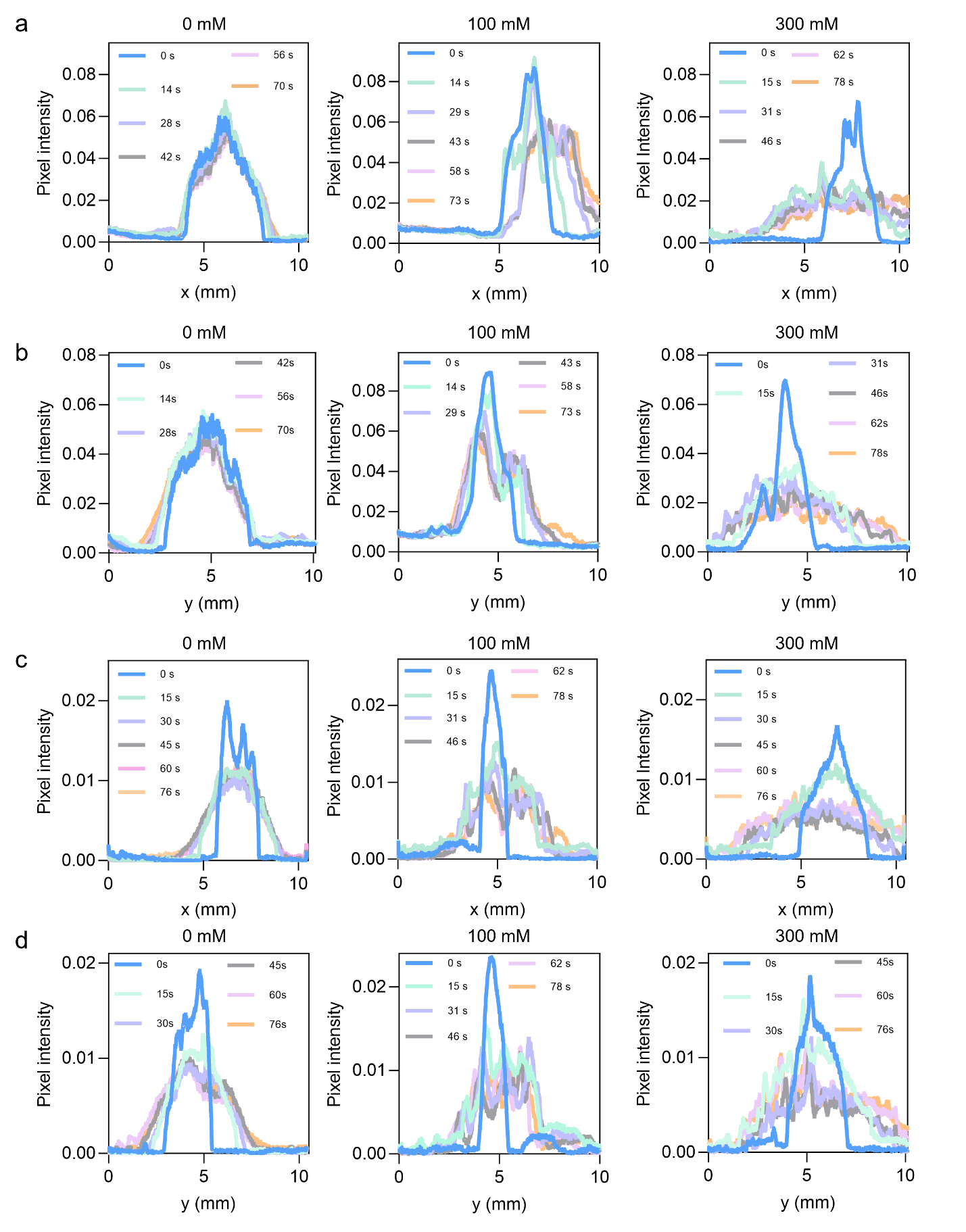


Figure S 9. Projections of the average pixel intensity along the a.) y-axis and b.) along the x-axis for NM in 0, 100 and 300 mM of urea for selected timepoints. Projections of the average pixel intensity along the c.) y-axis and d.) along the x-axis for NM@MMC in 0, 100 and 300 mM of urea for selected timepoints.


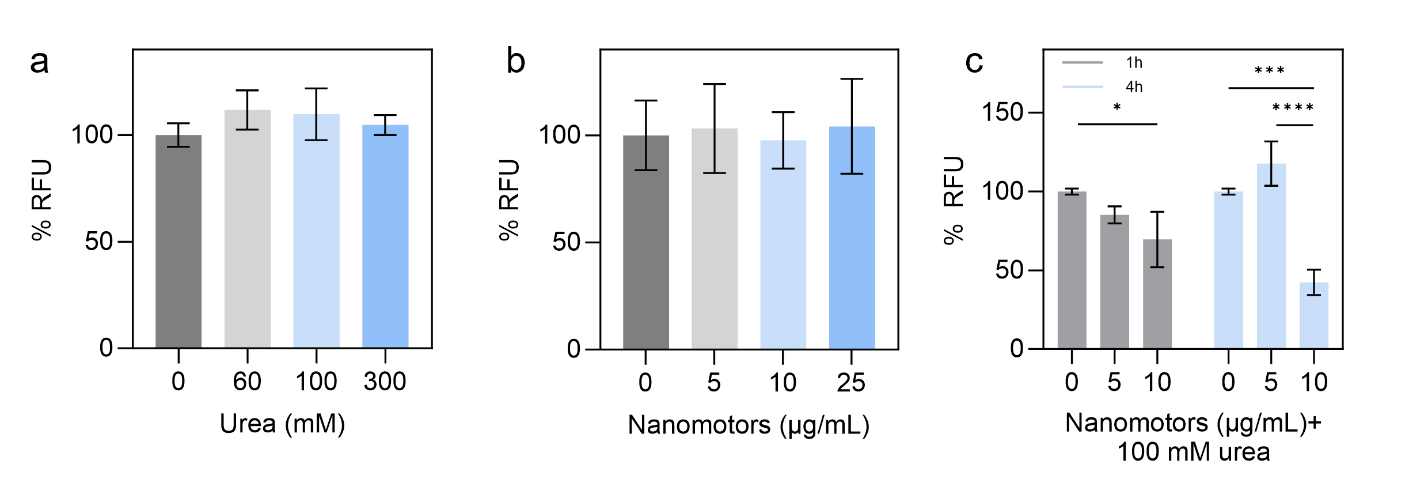


Figure S 10. Biocompatibility of NM components measured with Presto Blue cell viability reagent after 24 h of incubation with a) 0-300 mM urea and b) with NM at 0-25 µg/mL (n=3 biological replicates). c) Metabolic activity (% RFU) of MB49 cells after incubation with MSNP-NM at 5- and 10 µg/mL in presence of 100 mM urea after 1h and 4 h of incubation. The fluorescence intensity has been normalized with the average fluorescence intensity of the NT cells to obtain % RFU (n=3 biological replicates). Statistical significance (two-way ANOVA) is indicated when appropriate (*p < 0.05, **p < 0.01, ***p < 0.001, ****p < 0.0001).


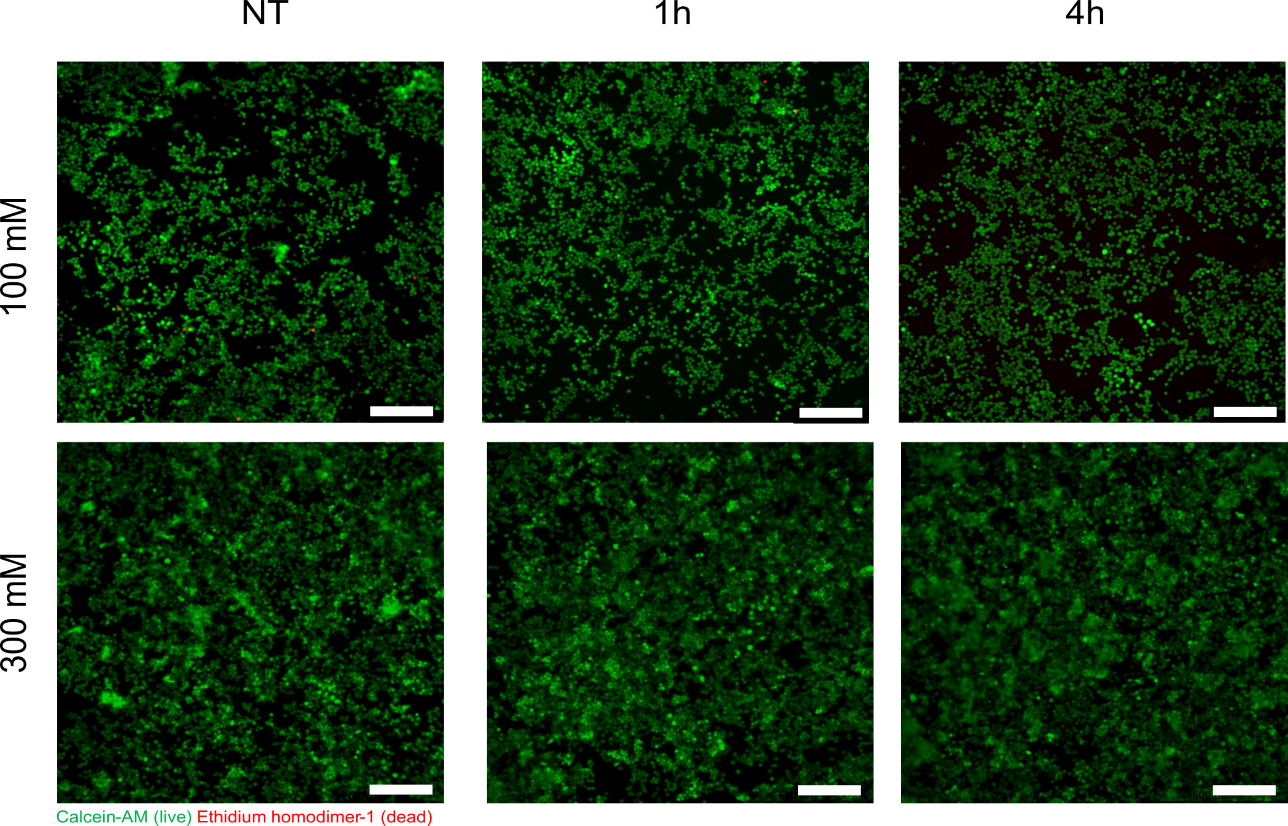


Figure S 11. LIVE/DEAD images of MB49 cells after incubation with 100 mM and 300 mM urea after 1h and 4h. The scale bar corresponds to 200 µm. Live cells are shown in green (Calcein-AM staining) and dead cells are shown in red (Ethidium homodimer-1).


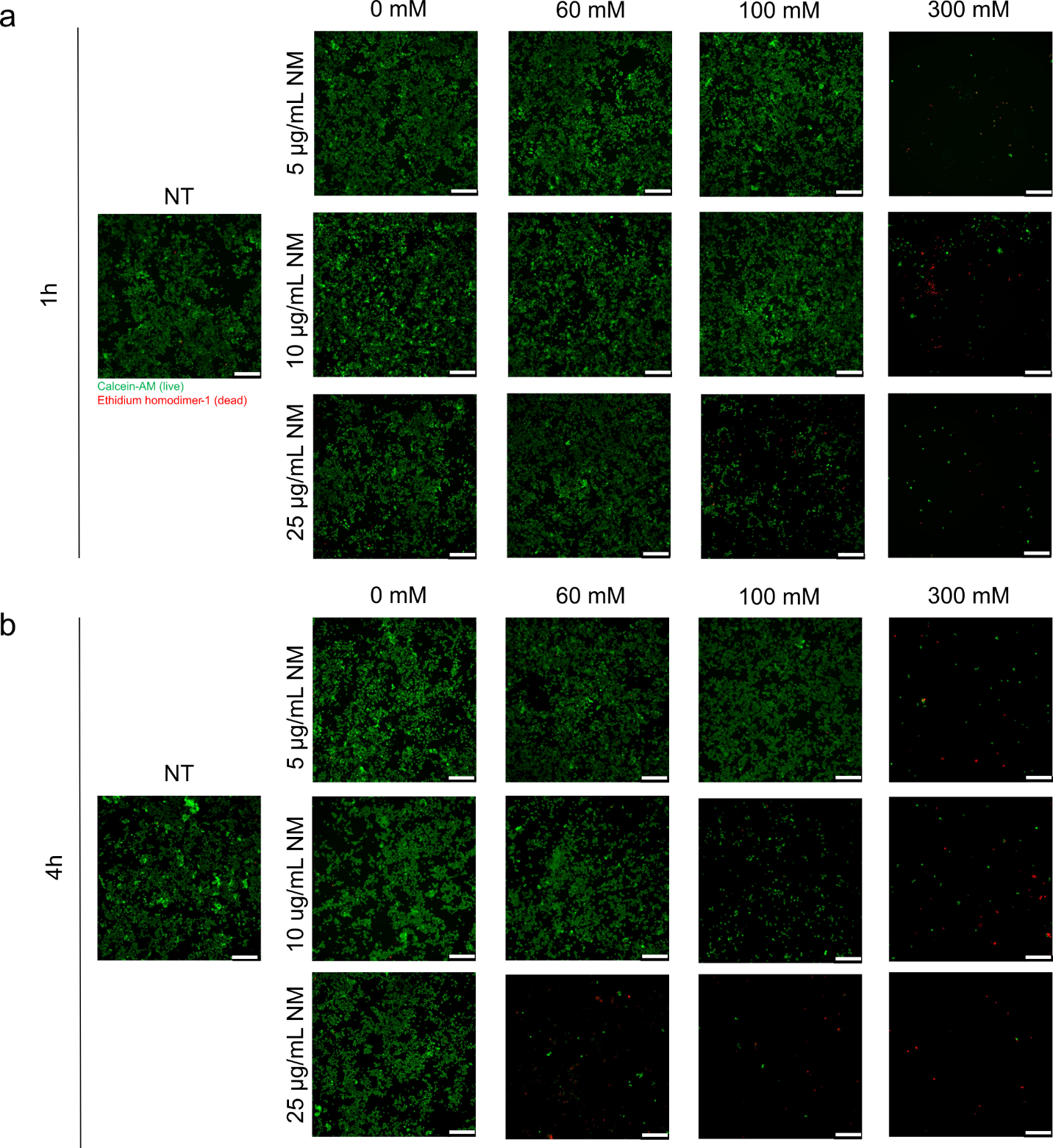


Figure S 12. Biocompatibility of urease-powered NM in presence of urea with MB49 cells based on LIVE/DEAD viability assay. LIVE/DEAD images of MB49 cells treated with Urease-NM at 5, 10 and 25 µg/mL after a.) 1 h and b) after 4h of incubation in presence of relevant concentrations of urea (0, 60, 100 and 300 mM). The scale bar corresponds to 200 µm. Live cells are shown in green (Calcein-AM staining) and dead cells are shown in red (Ethidium homodimer-1).


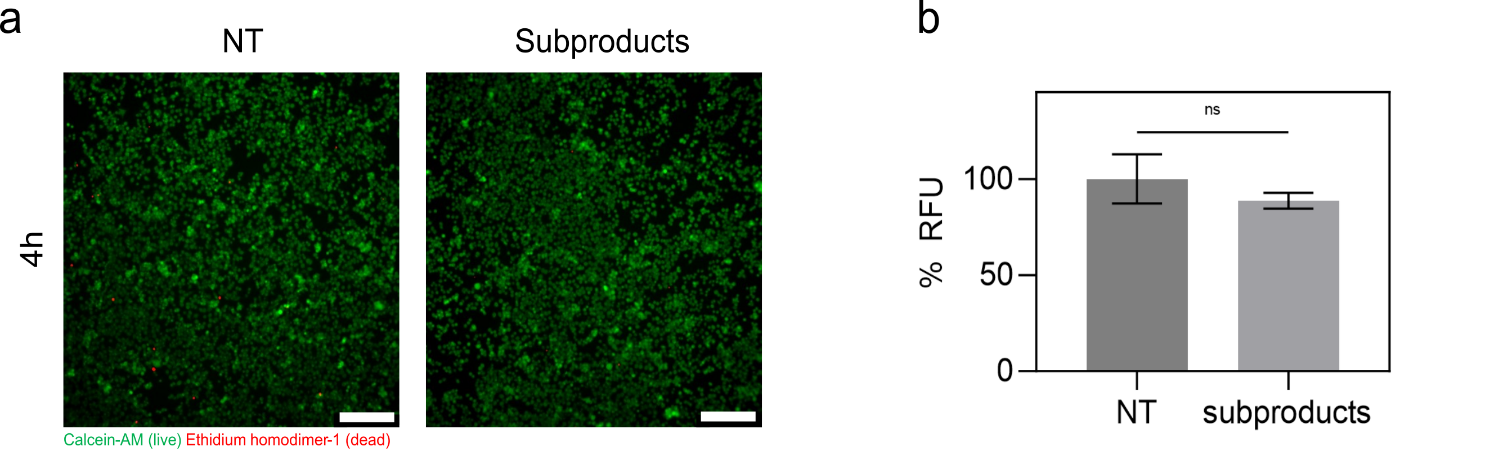


Figure S 13. Biocompatibility of subproducts of enzymatic reaction of nanomotors. a.) LIVE/DEAD images of MB49 cells after incubation with Subproducts generated by enzymatic reaction of 5 µg/mL Nanomotors with 100 mM urea. Cells were incubated for 4h with the subproducts before performing LIVE/DEAD imaging. The scale bar corresponds to 200 µm. Live cells are shown in green (Calcein-AM staining) and dead cells are shown in red (Ethidium homodimer-1). b.) Metabolic activity (% RFU) of MB49 cells after incubation with subproducts for 4h. The fluorescence intensity has been normalized with the average fluorescence intensity of the non-treated cells to obtain %RFU (n=3 biological replicates).


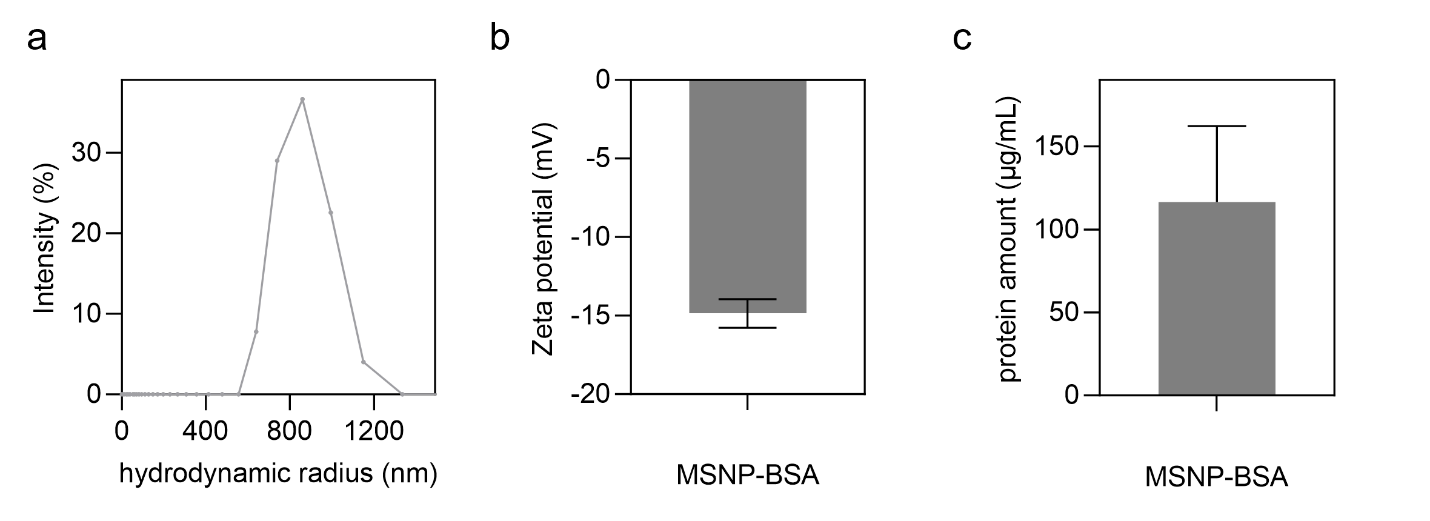


Figure S 14. Characterization of MSNP-BSA. a.) Hydrodynamic radius determined by light scattering and b.) Surface zeta potential and c.) protein amount bound to the MSNP.


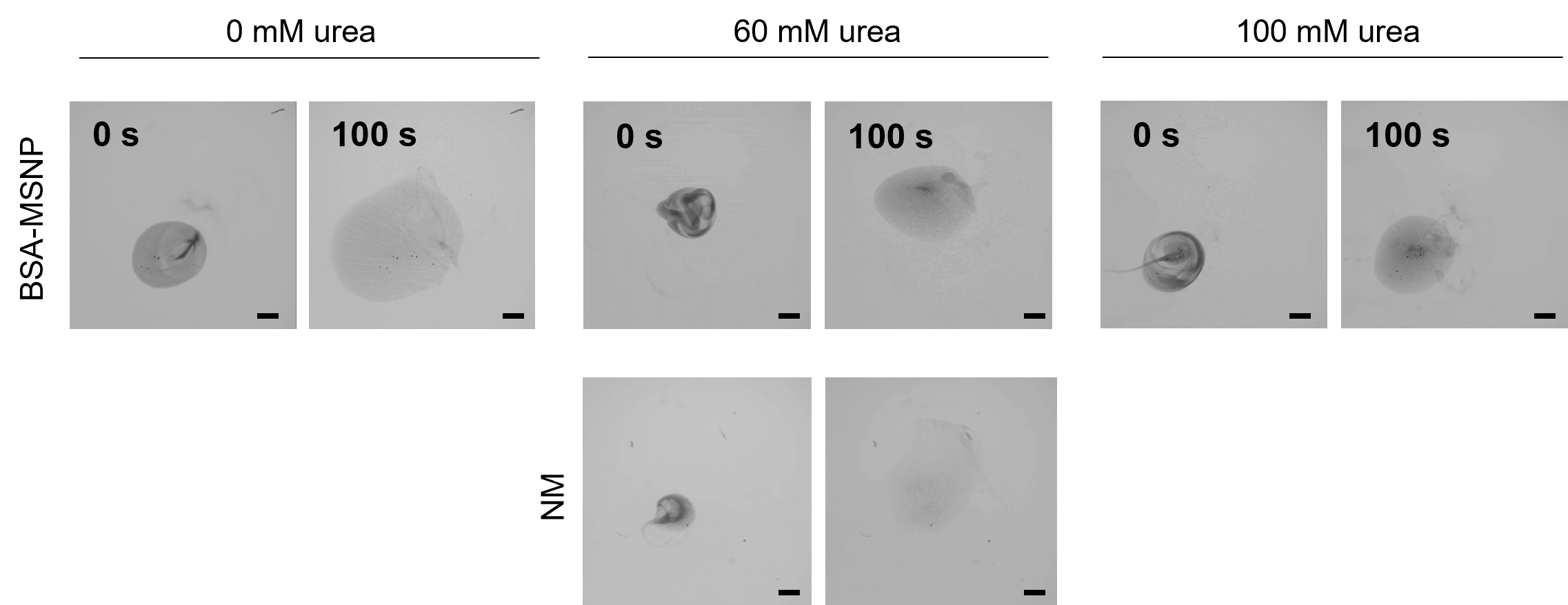


Figure S 15. Video snapshots of *in vitro* collective motion behavior of MSNP-BSA in presence of 0, 60 and 100 mM of urea and NM in presence of 60 mM urea. Snapshots represent NM swarms at 2 different time points (0s, 100s) taken from the top-view. The scale bar corresponds to 1 mm.


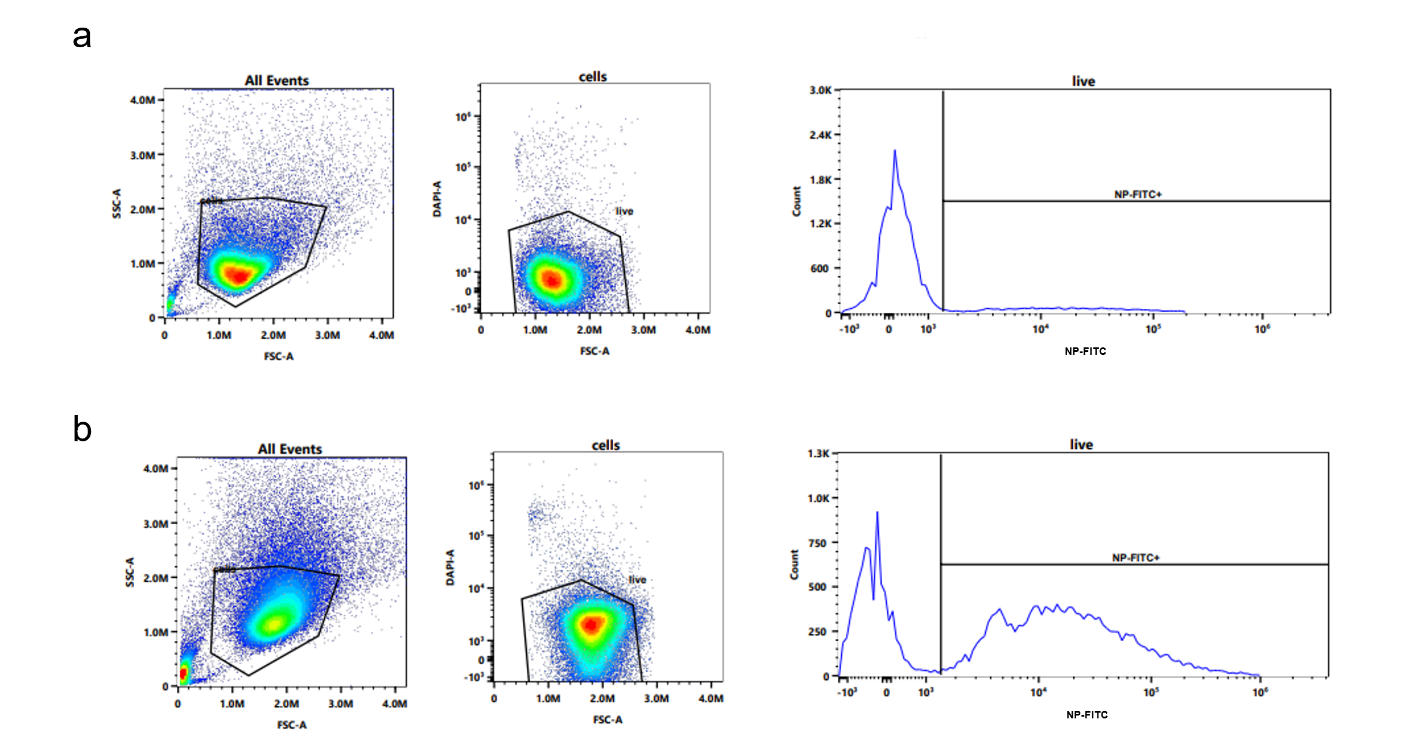


Figure S 16. Flow cytometry gating strategies. MB49 cell population gated based on their forward (FCS) and side scatter (SSC) properties, identification of live cells based on viability stain (DAPI) and identification of FITC-positive cells based on histogram analysis for a.) NM at 5 µg/mL and 0 mM urea and b.) NM at 5 µ/mL and 100 mM urea.


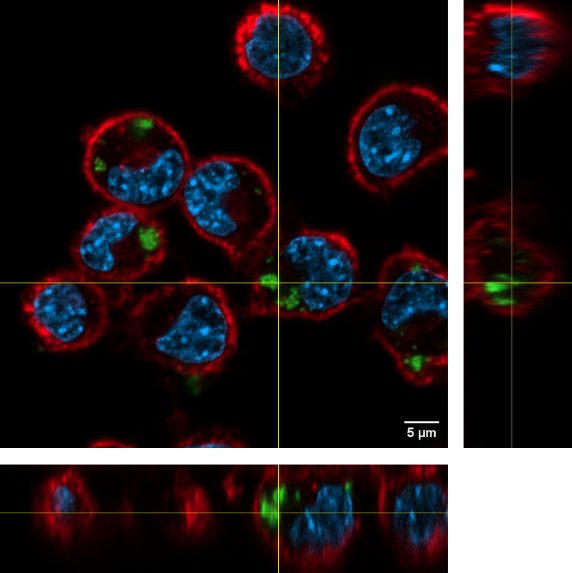


Figure S 17. Orthogonal view of confocal images showing MB49 cells with internalized FITC-labelled urease-powered NM (green) after treating the cells with 5 µg/mL NM in medium with 100 mM urea for 1h. Images were taken after additional 24h of incubation in cell medium. The cell membrane has been labelled with WGA (red) and the nucleus with Hoechst stain.


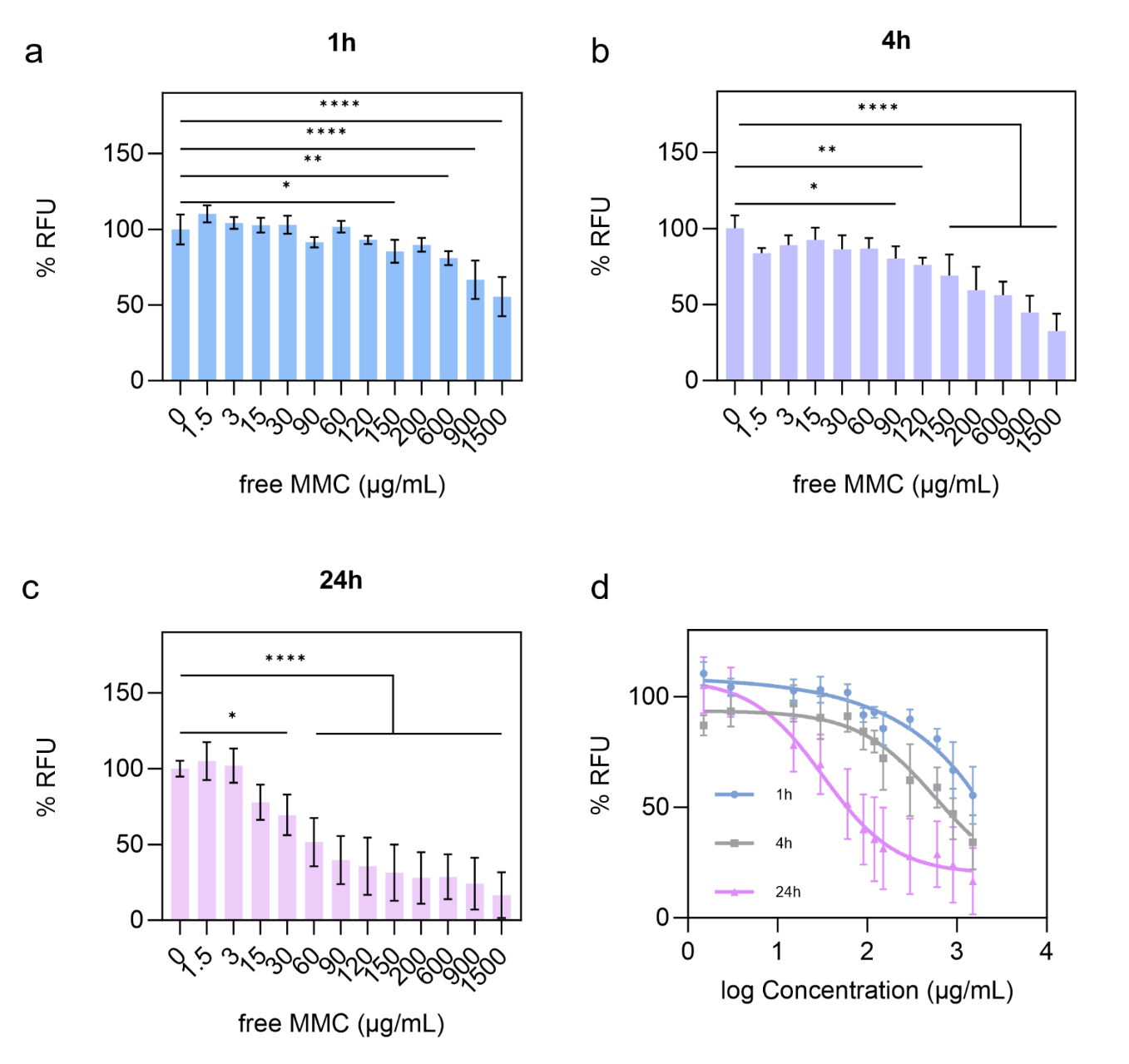


Figure S 18. Metabolic activity (% RFU) of MB49 cells after a.) 1 h b.) 4 h and c.) 24 h of incubation with free MMC. Metabolic activity has been determined by Presto Blue cell viability reagent. The results are represented as mean ± SD for n = 4 biological replicates. Statistical significance (one-way ANOVA) is indicated when appropriate (*p < 0.05, **p < 0.01, ***p< 0.001). d.) Representation of non-linear regression of the IC50 for MMC in MB49 cells after 1h, 4h and 24h of incubation.


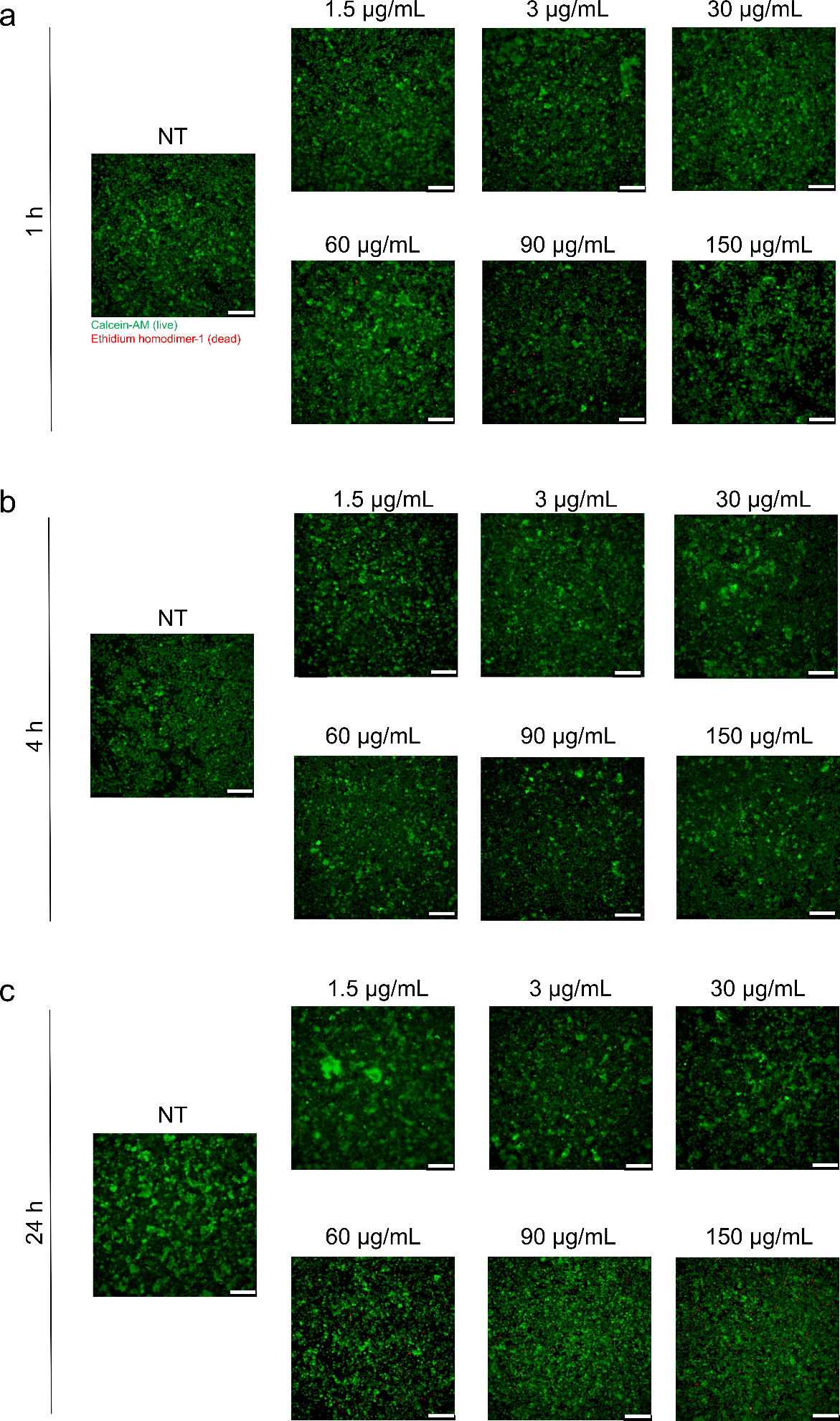


Figure S 19. LIVE/DEAD fluorescence images of MB49 cells incubated with free MMC for a.) 1 h, b.) 4 h and c.) 24 h of incubation. The scale bar corresponds to 200 µm. Live cells are shown in green (Calcein-AM staining) and dead cells are shown in red (Ethidium homodimer-1).


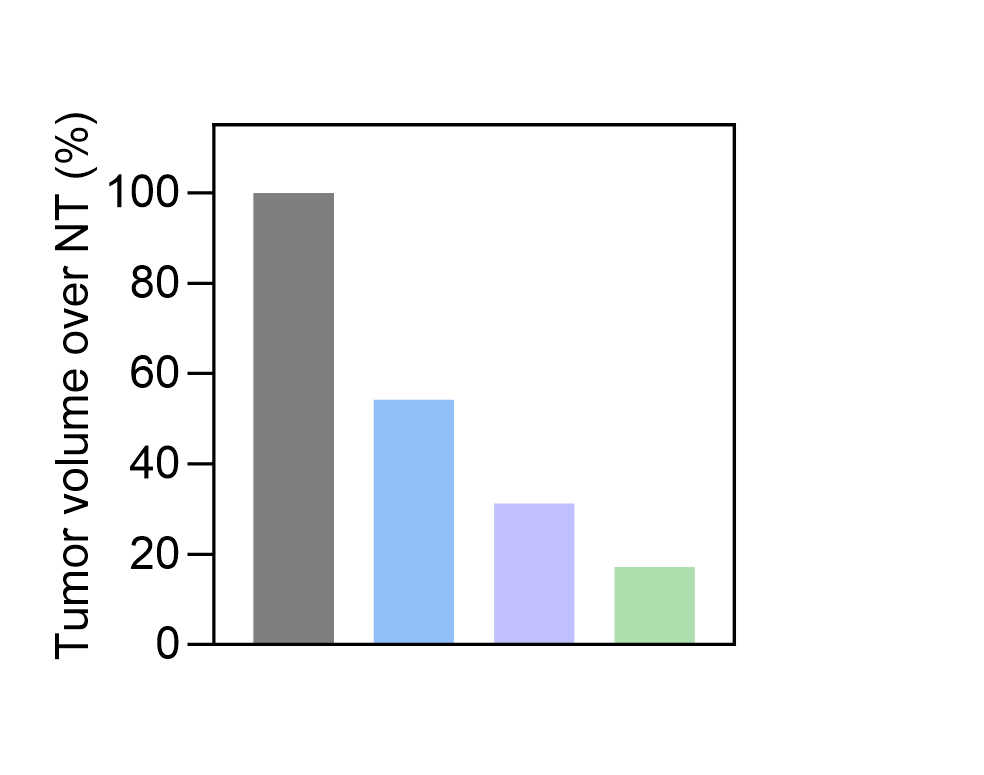


Figure S 20: Tumor volumes normalized over control condition (non-treated group) after 2 weeks post-treatment.


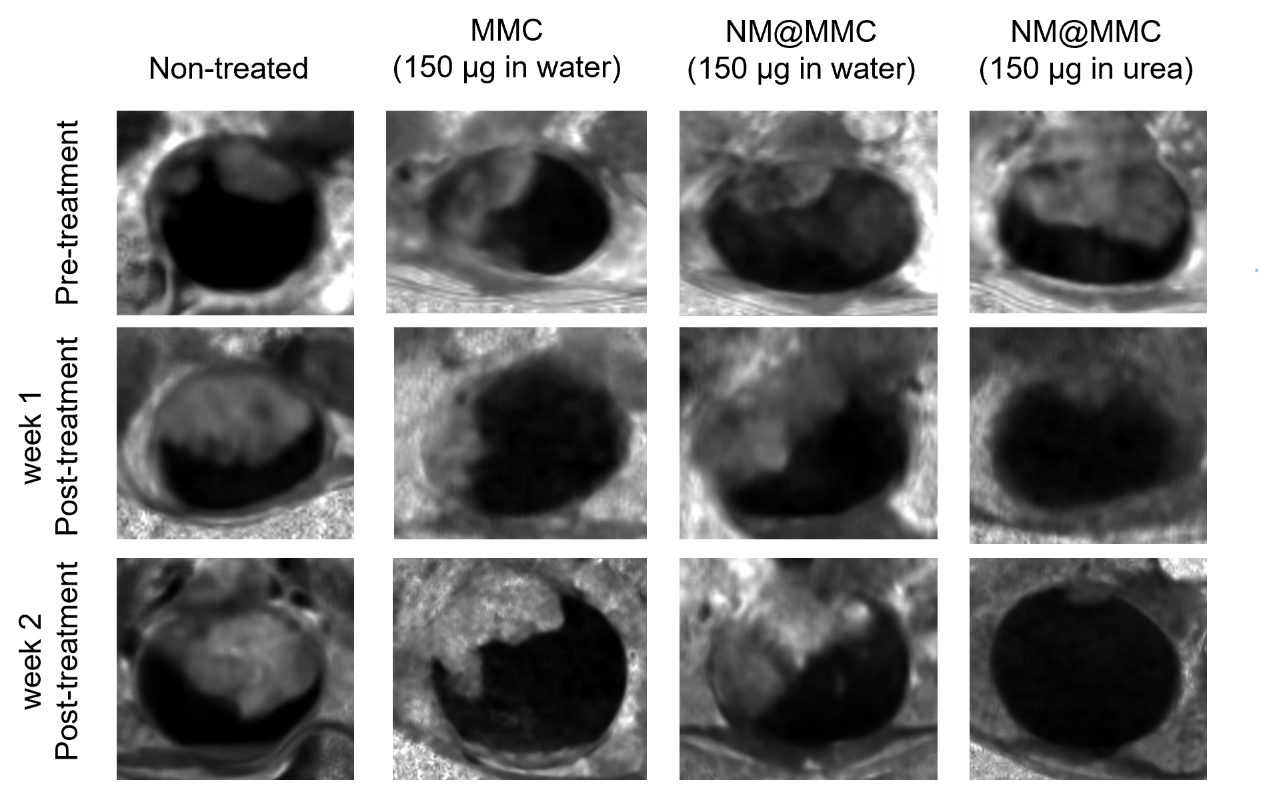


Figure S 21: Representative 2D DW-MRI images of the bladder (hypointense circular region) of representative mice before and after treatment.

**References Supporting Information.**

[1] Allan, D. B., Caswell, T., Keim, N. C., van der Wel, C. M., & Verweij, R. W. (2024). soft-matter/trackpy: v0.6.4 (v0.6.4). Zenodo. https://doi.org/10.5281/zenodo.12708864

[2] C. Simó, M. Serra-Casablancas, A. C. Hortelao, V. Di Carlo, S. Guallar-Garrido, S. Plaza-García, R. M. Rabanal, P. Ramos-Cabrer, B. Yagüe, L. Aguado, et al., *Nat Nanotechnol* 2024, *19*, 554.
